# Supplementary figures and images for: Local negative feedback of Rac activity at the leading edge underlies a pilot pseudopod-like program for amoeboid cell guidance
Source: PLoS Biol. 2023 Sep 25;21(9):e3002307. doi: 10.1371/journal.pbio.3002307 (PMC10553818; doi:10.1371/journal.pbio.3002307)

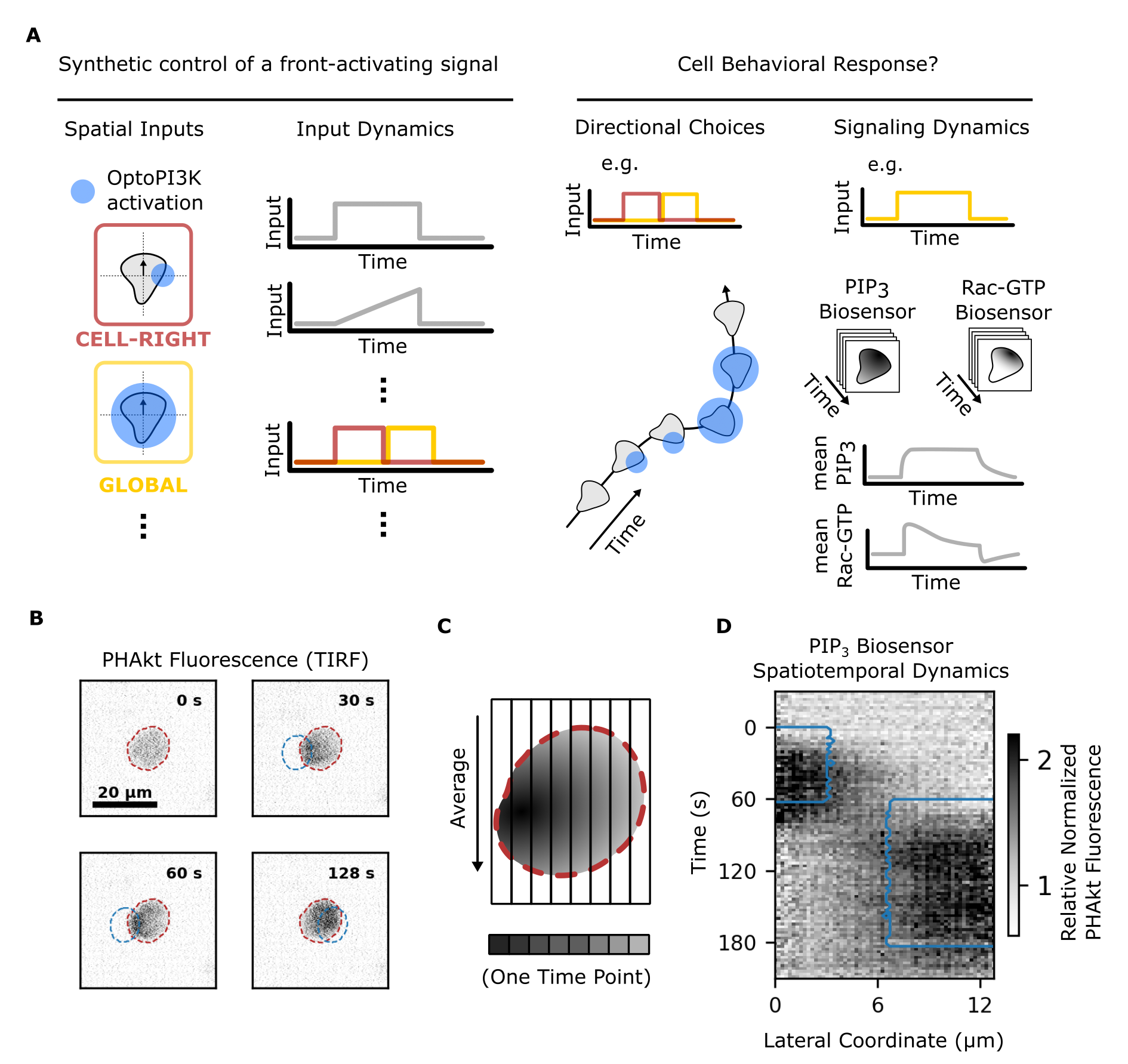

Supplement: S1 Fig — (A) To better understand how migrating cells make directional decisions, we used an optogenetic strategy for spatial and temporal control over PIP3, a signal that is sufficient to induce protrusion generation. Using TIRF microscopy, live-cell tracking, and computer vision–based automation, we applied various spatiotemporal dynamics of PIP3 generation to migrating cells and observed their signaling and migration responses. We used this strategy to identify the spatial and temporal features of input signals that control cell guidance. (B) TIRF microscopy of a 10-μM latrunculin-treated HL-60 cell that was exposed to blue light to activate opto-PI3K, first the left side and then the right side of the cell. (C) Schematic of quantification scheme. Background-subtracted pixel intensities within the segmented region (enclosed by red-dashed line) were binned based on their x position and then the average of each bin was collected to produce one vector per time point representing the average left-to-right fluorescence signal. (D) Kymograph of the PIP3 biosensor fluorescence signal (PHAkt-Halo (JF646)) of time and the lateral position of pixels (as quantified in S1C). The fluorescence signal closely corresponds to the dynamics of the blue light activation regions (enclosed within the blue outlines). The underlying data for this figure can be found in S1 Data. (TIFF) [file pbio.3002307.s001.tiff]

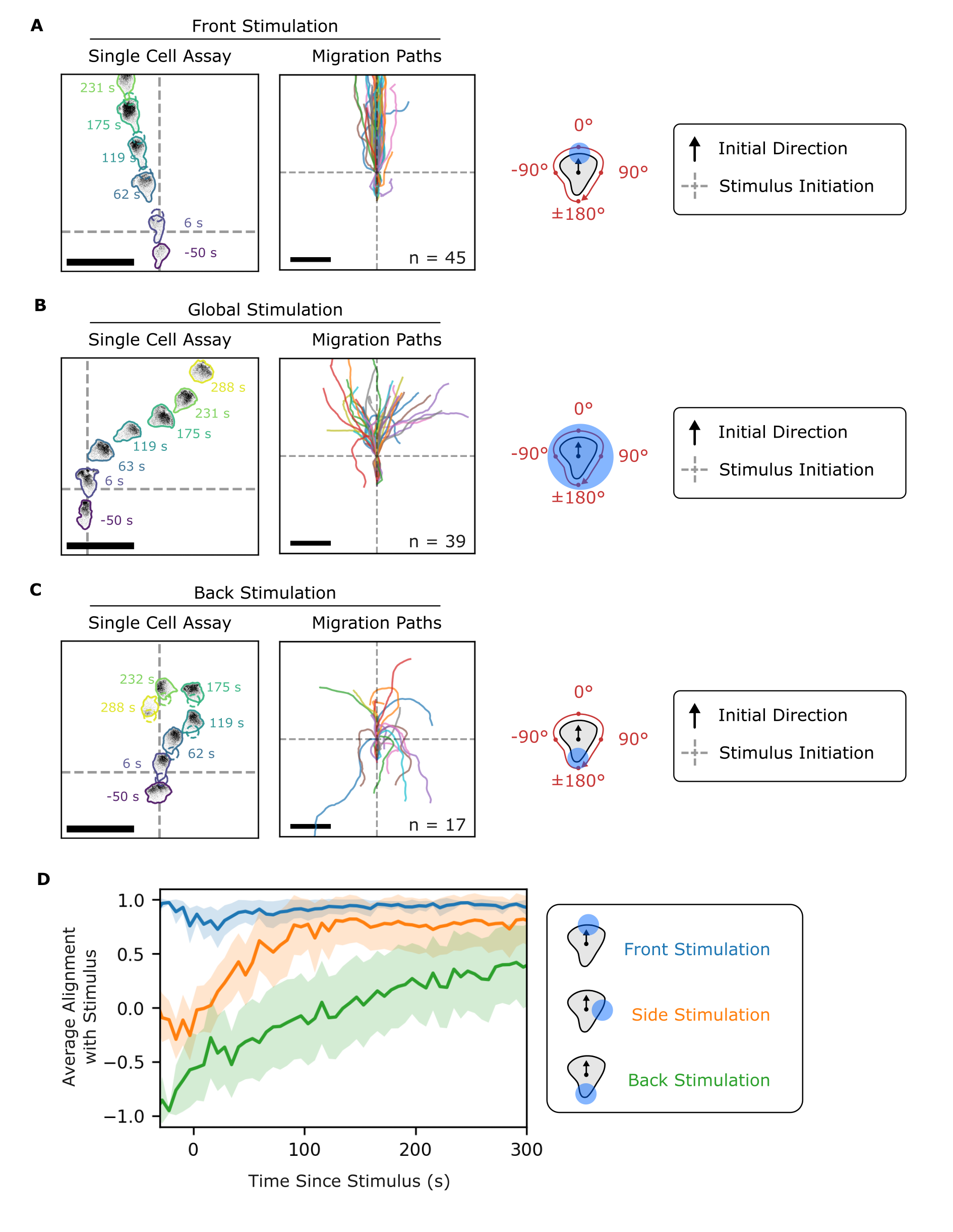

Supplement: S2 Fig — In each subpanel, the spatial data have been rotated and translated such that the cell displacement in the first minute of imaging (prestimulus) is toward the top of the figure, and the location of the cell at the zero second mark (moment of stimulation) is at the intersection of the gray, dashed lines. In each panel, Pak-PBD-mCherry (Rac biosensor) TIRF signal is shown in the Single Cell Assay subpanels. Scale bars in all subpanels: 50 μm (A) Migrating, opto-PI3K–expressing HL-60 cells were exposed to local blue light exposure at their fronts. The stimulus was centered at the cell edge at 0° relative to the displacement of the cell during the first minute of observation. The paths of 45 cells are shown (right). This pattern of stimulation causes hyperpersistent movement in the direction of stimulation. (B) Migrating, opto-PI3K–expressing HL-60 cells were exposed to uniform blue light exposure along their bottom surfaces. The paths of 39 cells are shown (right). This pattern tends to cause cells to deviate slightly to the right or left relative to their initial direction (see Fig 2E). (C) Migrating, opto-PI3K–expressing HL-60 cells were exposed to local blue light exposure at their backs. The paths of 17 cells are shown (right). This pattern of stimulation tended to cause cells to perform “u-turns.” (D) The angles of displacement for each cell were calculated, and cosine of the difference between these angles and the angle of stimulus were then calculated for each assay type as an indicator of stimulus alignment dynamics. Interestingly, back-stimulated cell alignment dynamics do not appear to match side-stimulated dynamics, even when they are spatially identical (i.e., when the green curve passes 0 on the y-axis). This is likely due to the spatial insensitivity at the cell back and the resulting slow local accumulation of PIP3 in this assay. Mean responses are shown with 95% CI shaded. The underlying data for this figure can be found in S1 Data. (TIFF) [file pbio.3002307.s002.tiff]

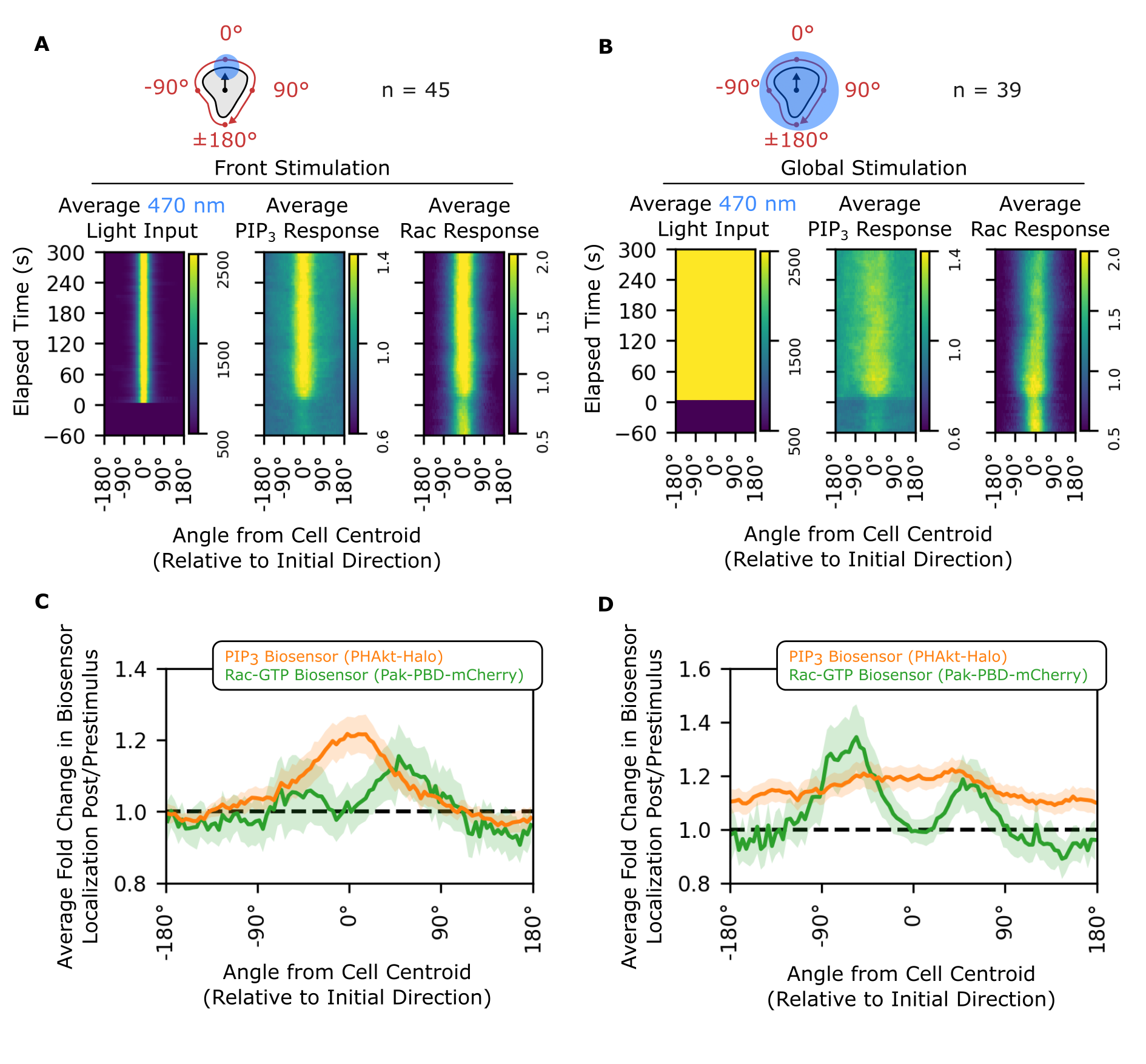

Supplement: S3 Fig — (Related to 2E) In panels A and B, a polar kymograph shows the average signal of interest around the segmented cell periphery over time during the course of an automated optogenetic stimulation protocol. (A) Local opto-PI3K stimulation at the fronts of migrating cells causes a localized increase in PHAkt-Halo localization (PIP3 biosensor) within the area of stimulation. Pak-PBD-mCherry (Rac biosensor) remains hyperpolarized throughout the course of the assay. (B) Global opto-PI3K stimulation causes a global increase in PHAkt-Halo (PIP3 biosensor) throughout the cell and a lateral spread in Pak-PBD-mCherry localization. Both signals stay relatively polarized near the 0° mark. (C) Fold-change in PIP3 and Rac reporters as a function of angle in the front-stimulated cell experiments. These are the means of single-cell ratios where each ratio is the average signal 1 minute poststimulus over the average signal 1 minute prestimulus. The PIP3 signal shows again that we have spatial control over our signaling input. The Rac signal shows evidence of saturation at the center of the fronts of cells (0°) but shows increases at the edges of the front. Mean responses shown with 95% CI shaded. (D) Similar to S3C, this panel shows the fold change in biosensors around the peripheries of cells during the first minute of stimulation. These curves show the mean responses of globally stimulated cells, which show the same saturation phenomenon at the fronts of cells (0°), while maintaining a capacity for increases at the edges of this front. Mean responses are shown with 95% CI shaded. The underlying data for this figure can be found in S1 Data. (TIFF) [file pbio.3002307.s003.tiff]

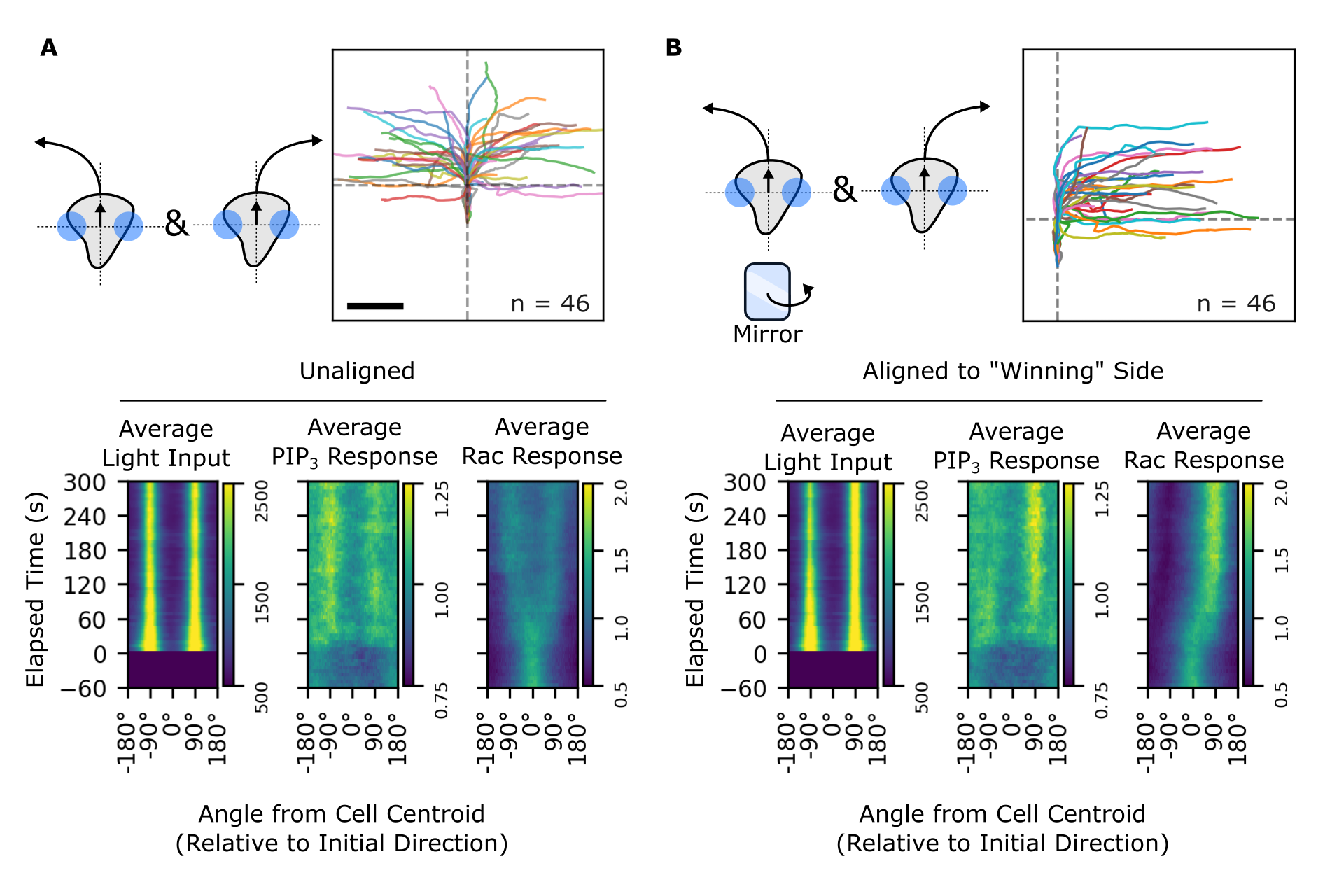

Supplement: S4 Fig — (A) When opto-PI3K–expressing cells are locally stimulated with blue light at ±90°, they stably orient toward only one of the two stimuli. Edge kymographs of averaged biosensor signals, as performed previously (Fig 1), are difficult to interpret in this assay. Therefore, we aligned cells to the “winning side” by horizontally reflecting cells that migrated left. (B) Edge kymographs of aligned cells show that we can locally produce PIP3 and that it is initially similar on both the “winning” and the “losing” sides. The data in Fig 2B come from the columns at ±90° in the Average PIP3 Response panel. The underlying data for this figure can be found in S1 Data. (TIFF) [file pbio.3002307.s004.tiff]

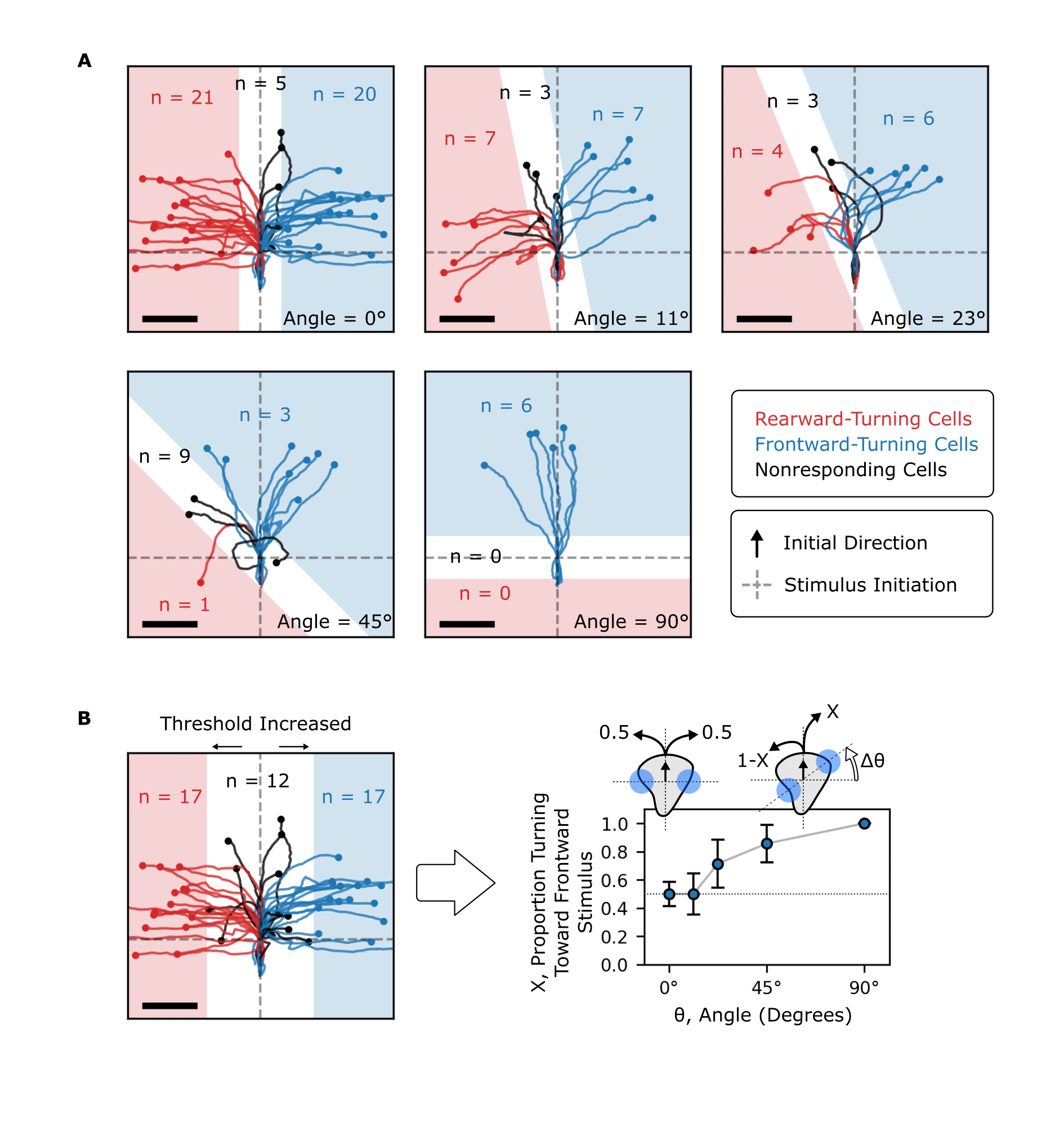

Supplement: S5 Fig — (A) Migration paths: For each tested stimulation angle, migrating cells were exposed to two sites of activation simultaneously. These sites were oriented at the indicated angle relative to the initial direction of movement. Those that had migrated 20 μm or more in the direction of the frontward stimulus at the end of the 5-minute assay were classified as moving frontward (blue lines), while those that migrated 20 μm or more in the direction of a rearward stimulus were classified as moving rearward (red lines). All other cells were classified as nonresponders (black lines). Shaded regions indicate the classification regions. Scale bars, 50 μm. (B) Robustness analysis: We chose to use 20 μm as our cutoff in Fig 2C, but the results hold for other distance cutoffs as well. For example, this shows the proportions as in Fig 2C, but with a 50-μm threshold for the classification rather than 20 μm. Scale bar, 50 μm. Proportions shown with 95% CI error bars. The underlying data for this figure can be found in S1 Data. (TIFF) [file pbio.3002307.s005.tiff]

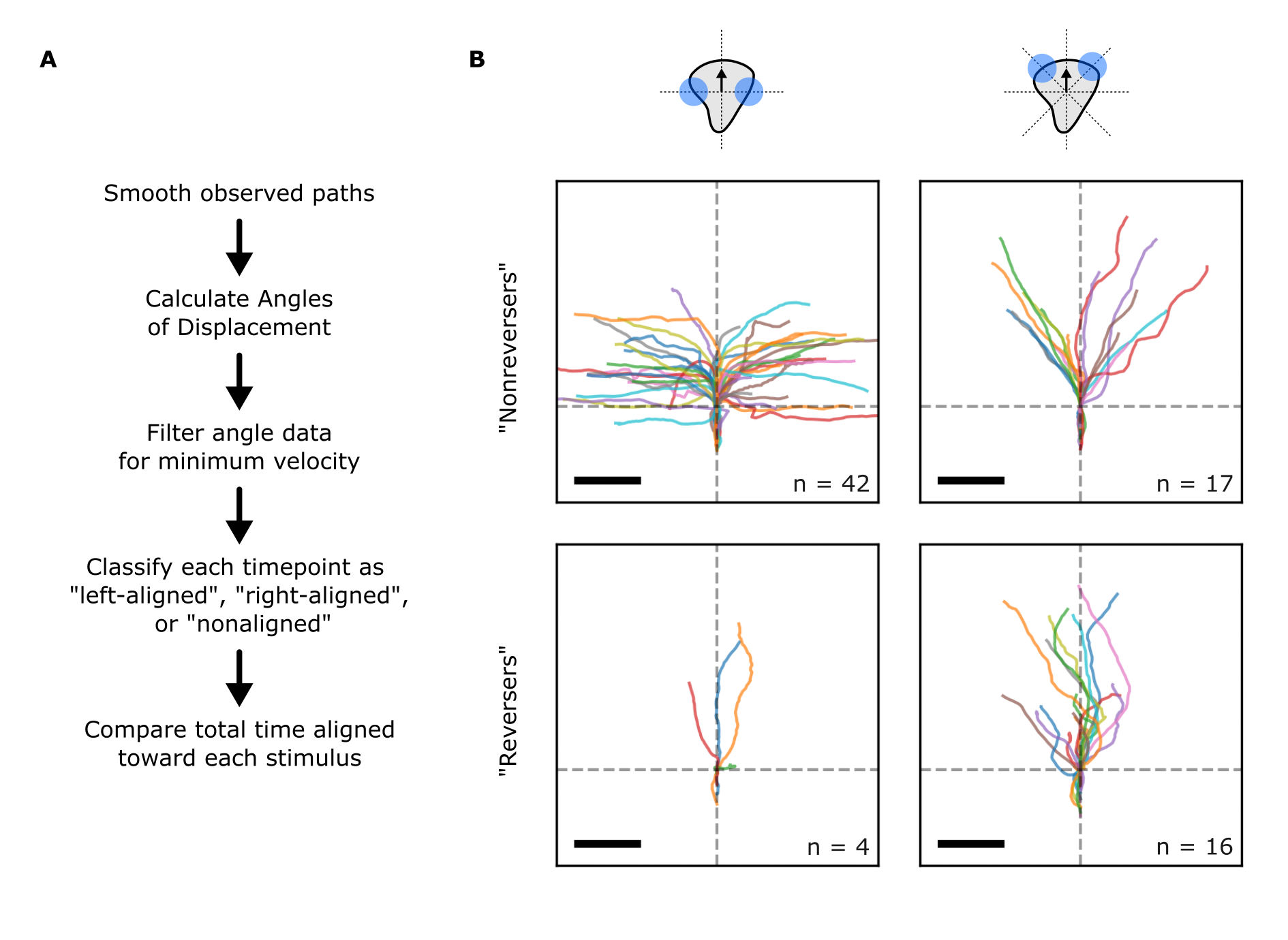

Supplement: S6 Fig — (A) Data processing pipeline for reversal classification. Full details are included in the methods section. (B) Migrating cells were stimulated with two local opto-PI3K stimuli located at either ±90° or ±45° relative to the initial direction of migration. Few cells stimulated with the ±90° pattern exhibit reversals. About half of cells stimulated with ±45° stimulus pattern exhibit reversals during the 5-minute assay. Scale bars, 50 μm. The underlying data for this figure can be found in S1 Data. (TIFF) [file pbio.3002307.s006.tiff]

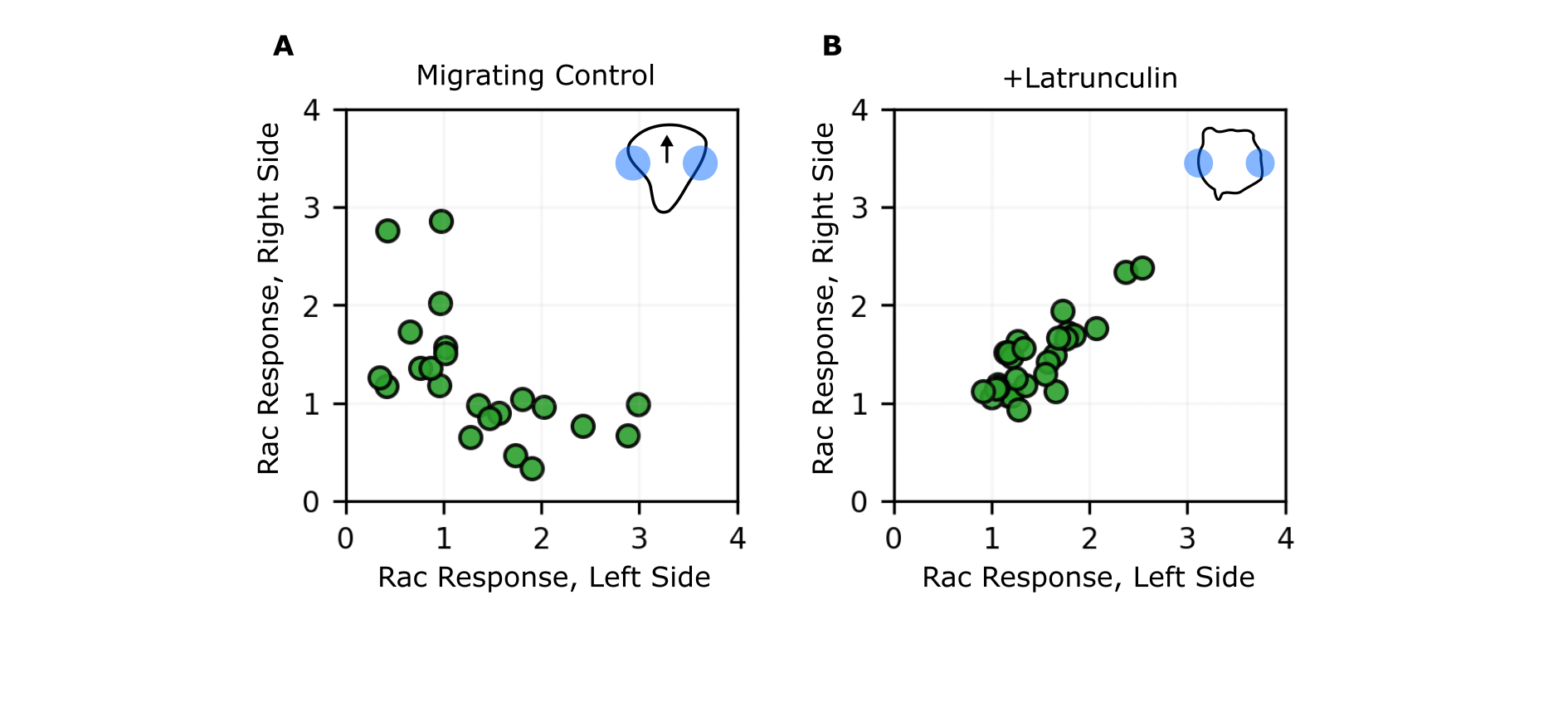

Supplement: S7 Fig — By comparing the Rac biosensor dynamics on the left and right sides of cells, we tested for the winner-take-all behavior expected in the presence of a global inhibitor. Each point is the relative Rac enrichment (compared to baseline) at the end of a 5-minute two-spot opto-PI3K activation assay. (A) In control cells stimulated with opto-PI3K at both their left and right edges (relative to their initial directionality), one site becomes Rac-high and one becomes Rac-low, leading to anticorrelated Rac distributions between the two sides. (B) In latrunculin-treated cells stimulated on both their right and left edges with opto-PI3K, this winner-take-all system is not functional, and the two sides now show a positive correlation of Rac activity levels. The underlying data for this figure can be found in S1 Data. (TIFF) [file pbio.3002307.s007.tiff]

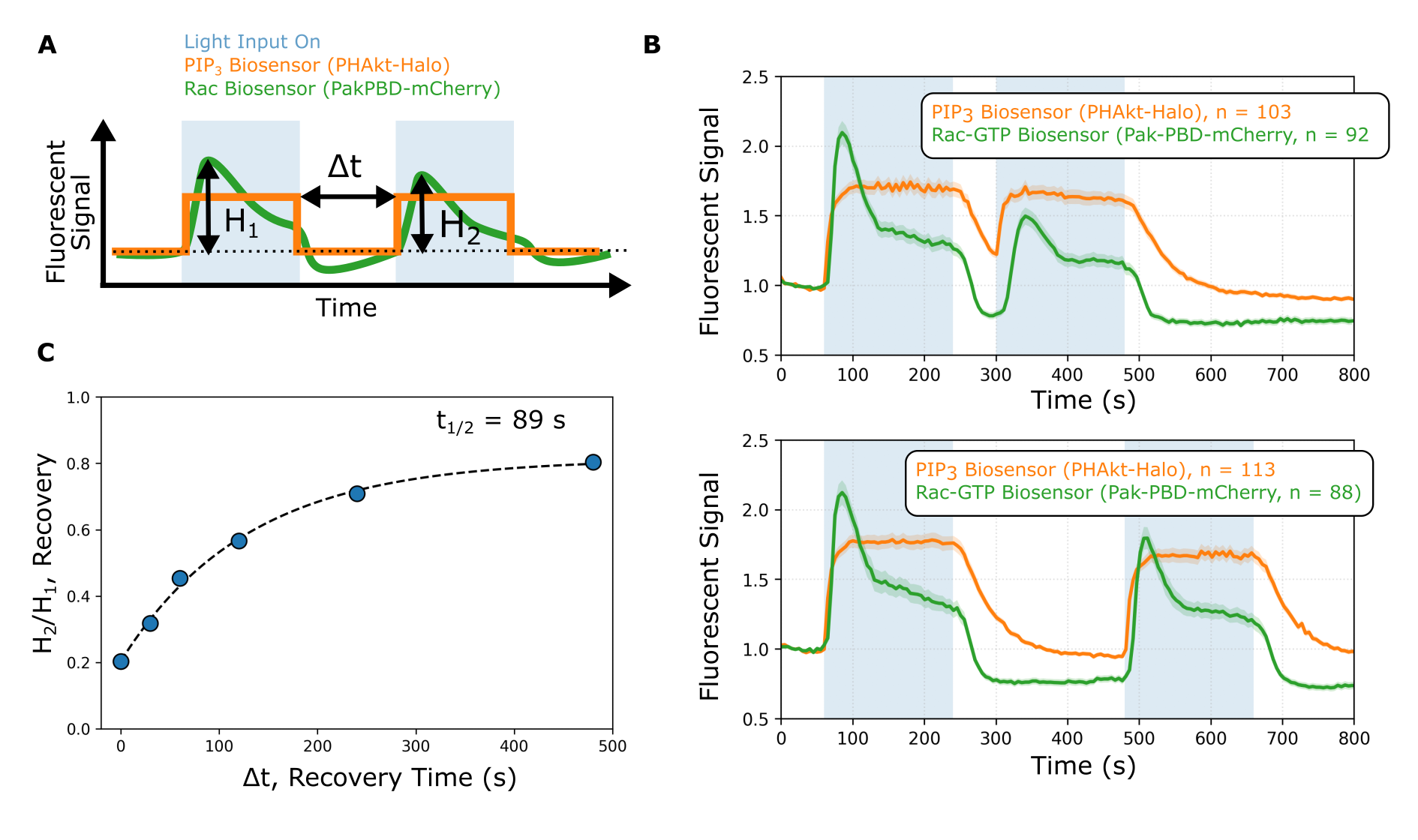

Supplement: S8 Fig — (A) Experimental schematic: Latrunculin-treated (10 μM) cells were exposed to two pulses of opto-PI3K activation separated by a variable amount of recovery time. We then calculated the ratio of the peaks heights for the first and second Rac responses. (B) Two example curves show the response of the PIP3 and Rac biosensors to two pulses of blue light activation, with recovery times of 60 seconds (top) and 240 seconds (bottom). Sample averages ± 95% CI are shown. (C) Degree of recovery as a function of recovery time. The Rac response has a recovery half-time of 89 seconds following opto-PI3K stimulation. The underlying data for this figure can be found in S1 Data. (TIFF) [file pbio.3002307.s008.tiff]

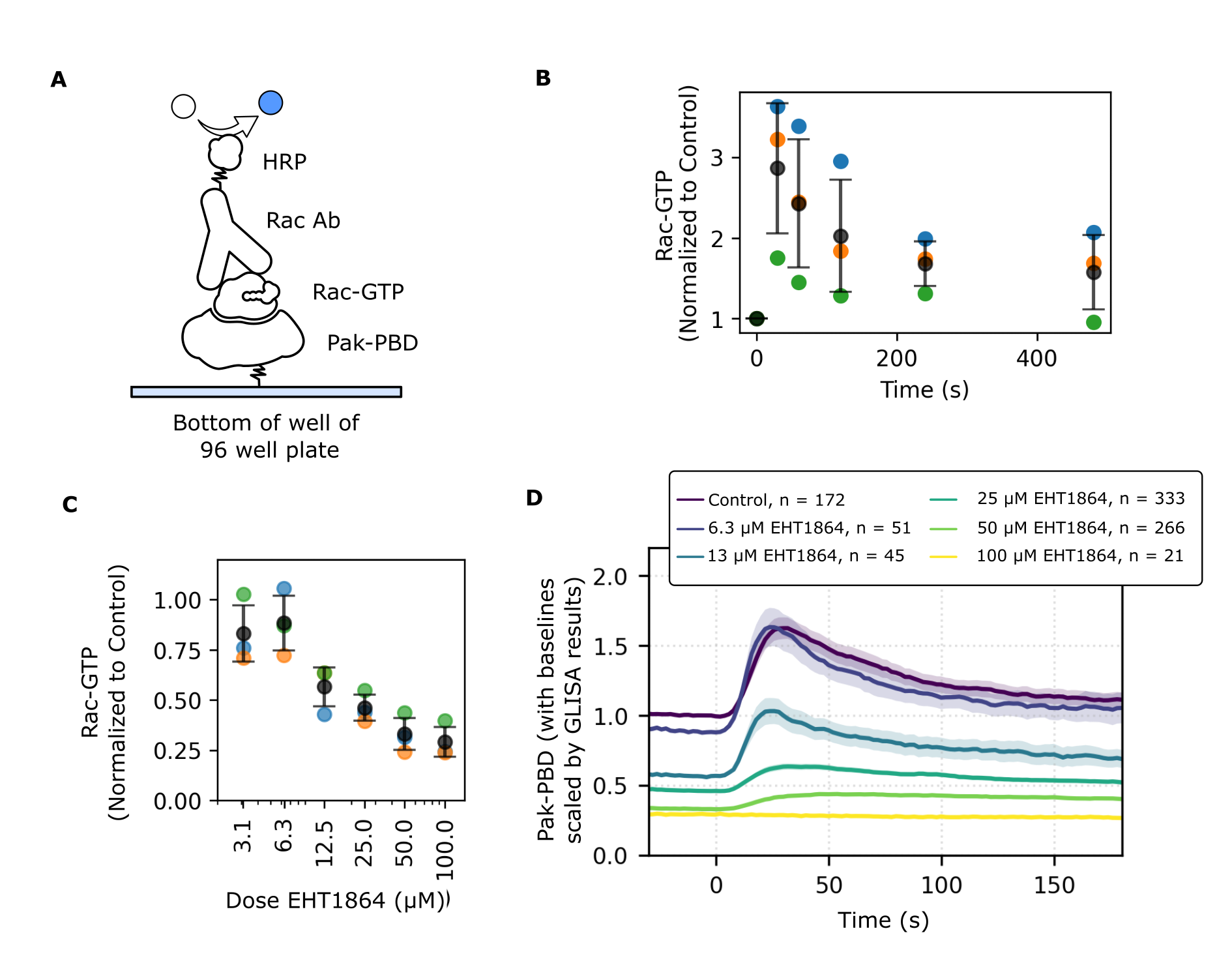

Supplement: S9 Fig — (A) Schematic of the ELISA-like mechanism used in the assay. Plate-bound Pak-PBD binds only the active form of cellular Rac (Rac-GTP). This bound active Rac can then be detected and quantified through a colorimetric assay using an HRP-conjugated Rac antibody. (B) GLISA-based measurements of relative Rac-GTP in blue-light-exposed opto-PI3K cells closely match the dynamics observed using the live-cell Rac biosensor, Pak-PBD-mCherry (compare with Fig 4A, left). Individual replicates are shown in blue, orange, and green. Sample averages ± SD are shown in black. (C) Dose–response showing proportion of active Rac in unstimulated opto-PI3K cells (as measured by GLISA) as a function of dose of EHT1864. Individual replicates are shown in blue, orange, and green. Means ± SD are shown in black. (D) Dose response of Pak-PBD dynamics in EHT1864-treated cells. Pak-PBD measurements were made via TIRF microscopy and normalized to baselines that were scaled by GLISA results from S9C. Mean responses are shown with 95% CI shaded. The underlying data for this figure can be found in S1 Data. (TIFF) [file pbio.3002307.s009.tiff]

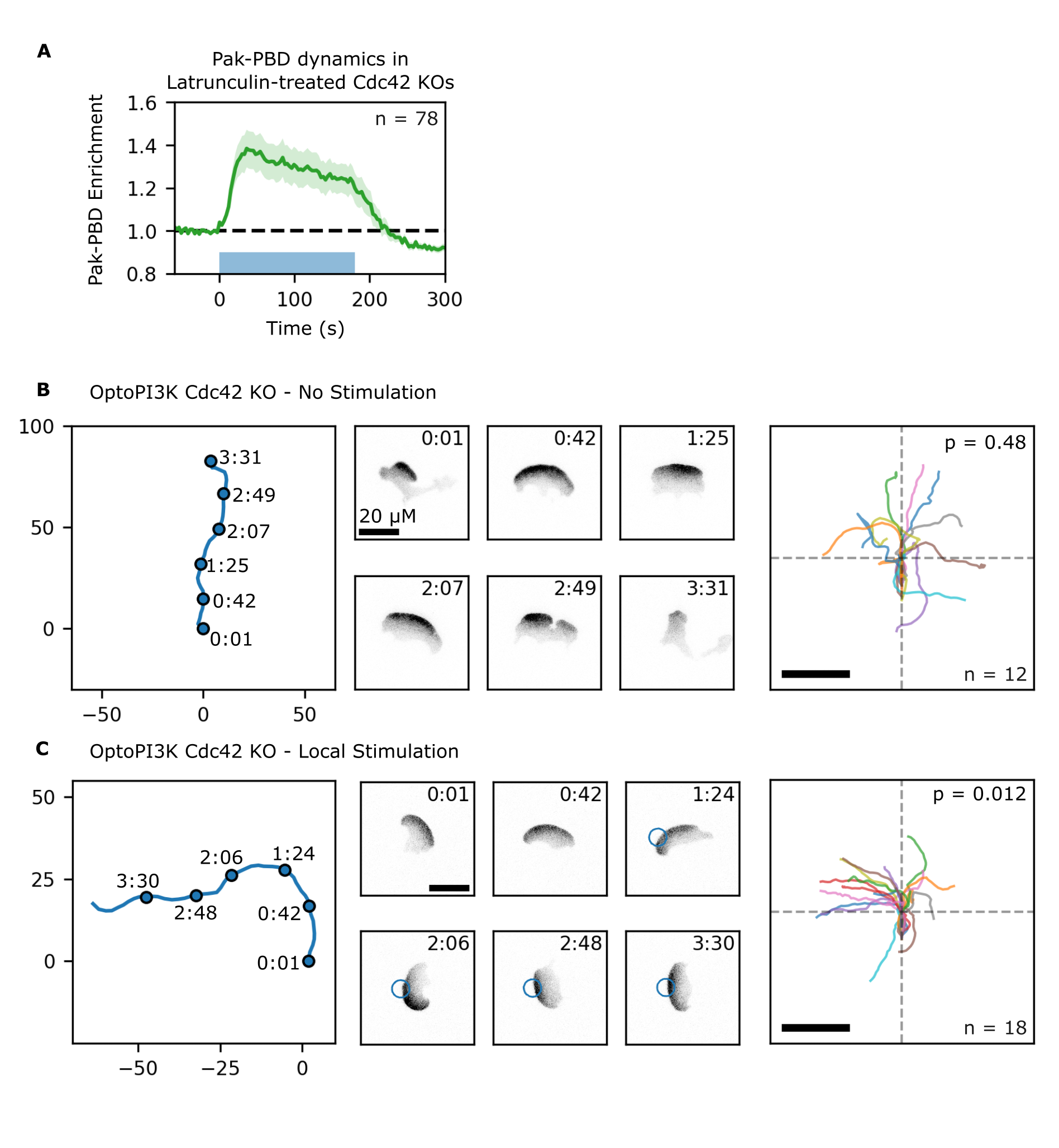

Supplement: S10 Fig — (A) Cdc42-null PLB cells exhibit adaptive recruitment of the Rac biosensor PBD following a step input of opto-PI3K, as in wild-type HL-60 cells (compare with Fig 4A, left). Opto-PI3K–expressing Cdc42-null cells were stimulated with blue light for 180 seconds, during which time they experienced a decline in the mean Pak-PBD TIRF localization signal. Mean response is shown with 95% CI shaded. (B) Cdc42-null cells maintain their ability to migrate and polarize Pak-PBD. The Cdc42-null cells have broad and relatively unstable fronts that appear to oscillate in width and frequently split. The p-value refers to the probability that the average final location of cells is unbiased (i.e., is equal to zero). In the absence of opto-PI3K stimulation, there is no bias. Scale bar for microscopy insets (center): 20 μm. Scale bar for migration paths (right): 50 μm. (C) In cells that maintain polarization during the course of optogenetic stimulation, local opto-PI3K stimulation retains its ability to steer Cdc42-null HL-60 cells. Cells were stimulated at 1:00 on their left side. The p-value refers to the probability that the average final location of cells is unbiased (i.e., is equal to zero). In the presence of a leftward opto-PI3K stimulus, there is a significant bias in cell directionality. Area of optogenetic activation shown with blue outline. Scale bar for microscopy insets (center): 20 μm. Scale bar for migration paths (right): 50 μm. The underlying data for this figure can be found in S1 Data. (TIFF) [file pbio.3002307.s010.tiff]

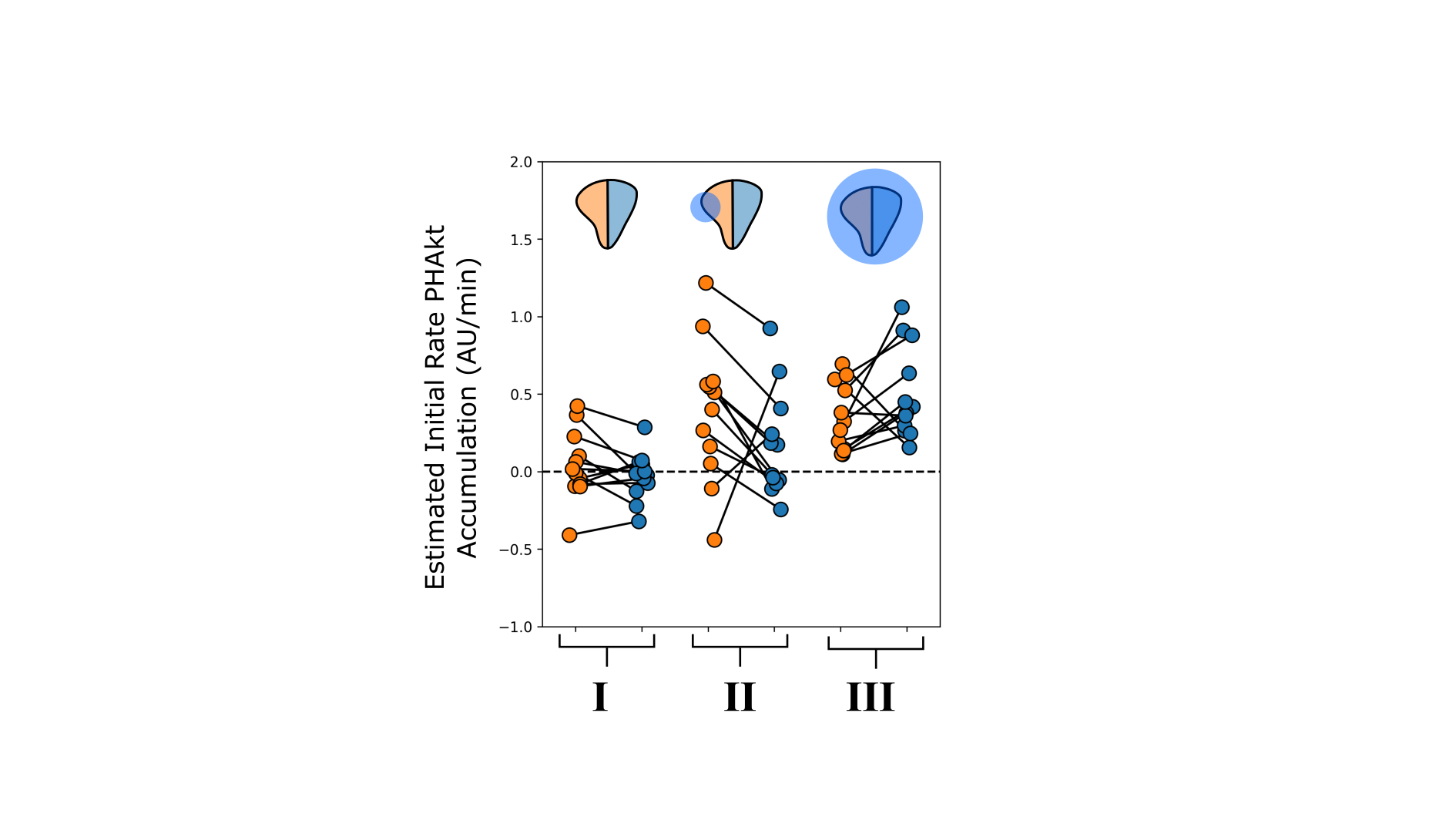

Supplement: S11 Fig — Differences in the estimated initial rates of PIP3 reporter (PHAkt-Halo) accumulation on the left and right sides of cells during the reversal assay (Fig 5). As expected, the majority of cells show similar PIP3 dynamics on their left and right sides prestimulation (phase I). However, cells show higher rates of PIP3 increase on the left side during the local stimulation phase of the assay (phase II) and higher rates of PIP3 increase on their right sides during the subsequent global stimulation phase of the assay (phase III). The underlying data for this figure can be found in S1 Data. (TIFF) [file pbio.3002307.s011.tiff]
