# Supplementary material for: Local negative feedback of Rac activity at the leading edge underlies a pilot pseudopod-like program for amoeboid cell guidance
Source: PLoS Biol. 2023 Sep 25;21(9):e3002307. doi: 10.1371/journal.pbio.3002307 (PMC10553818; doi:10.1371/journal.pbio.3002307)
Supplement: Attachment — Submitted filename: plos_revisions_response.pdf [file pbio.3002307.s020.pdf]

Detailed point-by-point response to the Reviewers

REVIEWS:

Reviewer #1: In this manuscript, Town and Weiner have probed into the decision-making strategy in migrating neutrophil-like HL-60 cells in response to multiple chemoattractant cues. Being the body's first line of defense, neutrophils need to polarize their cytoskeletal regulators toward chemical signals arising from injury and infection. Previous studies have shown that such polarization is brought about by local positive feedback that amplifies the cell front and global negative feedback which allows fronts to compete for dominance and enables the cell to persistently move in a single direction.

Although this model provides important insight on how cells establish polarity initially, it could not explain how cells can reorient or correct polarity to changing external cues. In this manuscript, the authors have tried to understand how migrating cells balance decisiveness with flexibility in respect to responding to chemoattractant cues. To mimic cellular responses to multiple cues at the same time, authors have employed two-spot optical competition assays using opto-PI3K to acutely increase PIP3 levels at different parts of the cell membrane simultaneously. Their results show that migratory responses varied spatially in polarized cells; in the presence of 180 degrees-opposed stimuli at the lateral edges of the existing front, cells migrated stably in either direction. Thus, they concluded that the front was more responsive to PI3K activation than the back. Once activation spots were moved to 45 degrees from the polarity axis to ensure stimulation would be continuously near the cell fronts, a high percentage of cells showed some degree of reversal between the two orientations. This orientation switching suggested that a local inhibitor at the front could be destabilizing protrusions, as predicted earlier by Meinhardt. The authors then moved ahead to isolate the effects of local inhibition by pharmacologically blocking activation of mechanically-gated global inhibition. Their results showed that local PI3K activation results in recruitment of a reversible and locally acting Rac inhibitor. Finally, the authors suggest that Rac activity is needed for its inhibition which is consistent with Rac-dependent negative feedback.

Overall, this study is technically impressive, however, there are significant gaps that need to be addressed before their main conclusions can be fully supported. In addition, the authors must do a much better job distinguishing the novelty of their findings, given that previous studies (mentioned in the text) have explored the existence of Meinhardt-style inhibitors in cell motility.

We thank the reviewer for their support. We have taken their comments to heart, and our revision buttresses the key points they raise that are important to support the major conclusions of the paper. We have also adjusted the introduction and discussion to more clearly emphasize the novelty of our work in connecting Meinhardt-style local inhibition to a modified pilot pseudopod model of cell guidance.

Major concerns:

1. The authors need to show that there is complete absence of global inhibition in presence of latrunculin. A major advancement that the authors claim in their manuscript is that they were able to delineate local inhibition by eliminating global inhibitory effects in migrating cells.

Great point. Based on previous work in which the global inhibitor is thought to depend on actin-protrusion-based membrane tension increases, we would expect that latrunculin treatment should abolish global inhibition, but it is true that our initial submission did not directly demonstrate this to be the case. We now formally show that latrunculin treatment breaks global inhibition. In latrunculin-treated cells, local opto-PI3K-mediated Rac activation is not affected by a secondary site of opto-PI3K activation (**Current Fig 3C**). Additionally, whereas control cells exhibit competition between two sites of opto-PI3K activation (**Current Fig S7A**), latrunculin-treated cells show no competition (**Current Fig S7B**).

3C

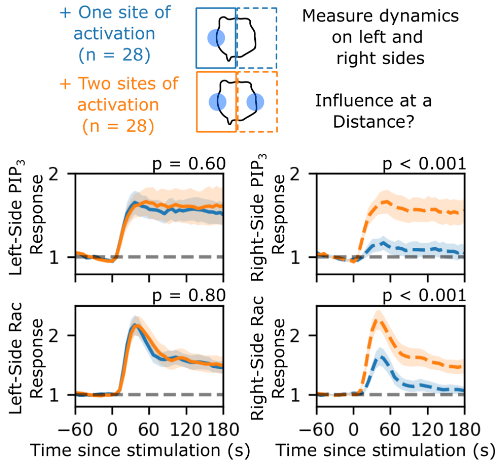

S7A

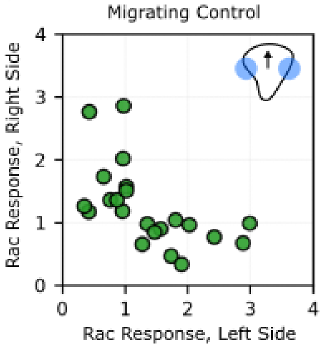

S7B

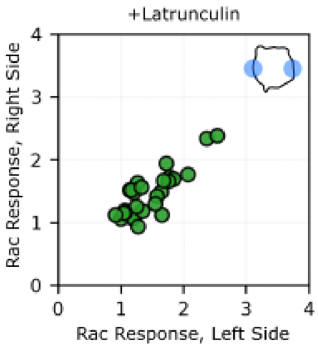

(3C) To demonstrate the absence of global inhibition in latrunculin-treated cells, we exposed them to either one or two spots of opto-PI3K activation and compared the resulting dynamics on both sides. In the absence of a global inhibitor, the responses for a single opto-PI3K stimulus should be the same whether or not there is a secondary site of opto-PI3K activation (in contrast to competition between these sites in the presence of the global inhibitor, as in Fig 2A-C). When the right sides of cells are compared in each type of stimulation, the presence or absence of the local right stimulus makes a large difference in the observed signaling dynamics. However, the dynamics on the left sides of cells do not differ based on the presence or absence of a distant site of stimulation. Thus, there is no inhibitory action-at-distance, which would be expected from a global inhibitor. The p values above each graph indicate the probability of observing an absolute difference greater than or equal to that observed by a permutation test. Mean local responses are shown with 95% CI shaded.

(S7) By comparing the Rac biosensor dynamics on the left and right sides of cells, we tested for the winner-take-all behavior expected in the presence of a global inhibitor. Each point is the relative Rac enrichment (compared to baseline) at the end of a 5-minute two-spot opto-PI3K activation assay. **(A)** In control cells stimulated with opto-PI3K at both their left and right edges (relative to their initial directionality), one site becomes Rac-high and one becomes Rac-low, leading to anticorrelated Rac distributions between the two sides. **(B)** In latrunculin-treated cells

stimulated on both their right and left edges with opto-PI3K, this winner-take-all system is not functional, and the two sides now show a positive correlation of Rac activity levels.

2. The authors claim that local inhibitor works solely on Rac. However, there was no evidence that molecules upstream of Rac, such as PI3K, were inhibited also. In Fig 3C, it is possible that PIP3 is being locally degraded at the first stimulation site.

We had hoped to simplify the presentation of the story by focusing on whether the observed inhibition was local or not (**Previous Fig 3**) before exploring possible mechanisms (**Previous Fig 4**). The Reviewer makes a good point however that omitting the PIP_3 biosensor data in Fig. 3 (**Previous Fig 3**) made this experiment difficult to interpret. We now provide PIP_3 polarization data in Fig. 3D (**Current Fig 3D**) to show that local inhibition is operating at the level of Rac activity (which overshoots) and not at the level of PIP_3 accumulation (which returns to baseline without overshooting).

3D

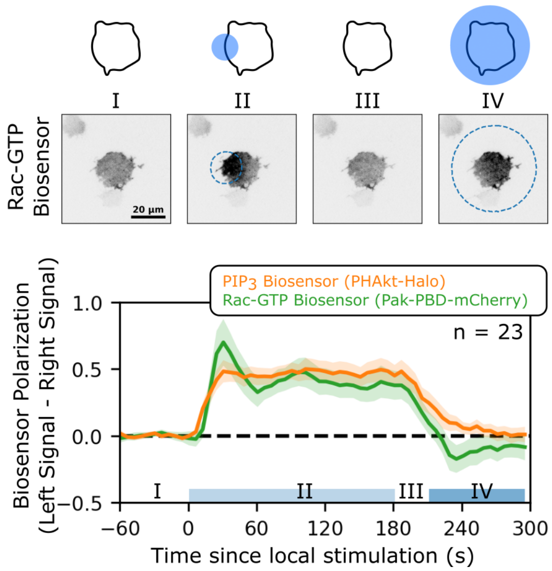

(3D) To demonstrate the presence of local inhibition, we first exposed single cells to a local opto-PI3K stimulus on their left sides, then allowed cells to recover briefly, and then exposed them to a global opto-PI3K stimulus. We reasoned that if the decline in signaling on the left side were due to a local inhibitor, the impact of that inhibition should be observable via a weaker response on the prestimulated side. We observed this evidence of local inhibition at the level of Rac activation (green – Rac is biased towards the right side in phase IV) but not at the level of the opto-PI3K input (orange – no bias of PIP_3 to the right side in phase IV). Mean local responses are shown with 95% CI shaded.

3. There is very little primary microscopy data in the main figures. This proved to be a hindrance in grasping the main findings of the manuscript. For example, Figure 1 contains many

illustrations which could be moved to a supplement to provide room for more actual data. Additionally, the microscopy images in Fig 2C and 5B are far too small to evaluate effectively.

Great suggestion. We have moved the schematics describing the experimental conceptualization and setup into the supplement (**Fig. S1A**) to make room for a proof-of-concept microscopy-based figure demonstrating our ability to dynamically spatially control PIP_3 production with light (**Current Fig. 1E** and **1F**). Similar analyses (**Current Fig S5**) should also help alleviate the reviewer concerns described in point 4 below.

Additionally, we have made extra effort to refer readers to supplemental movies which display the microscopy data in a larger format.

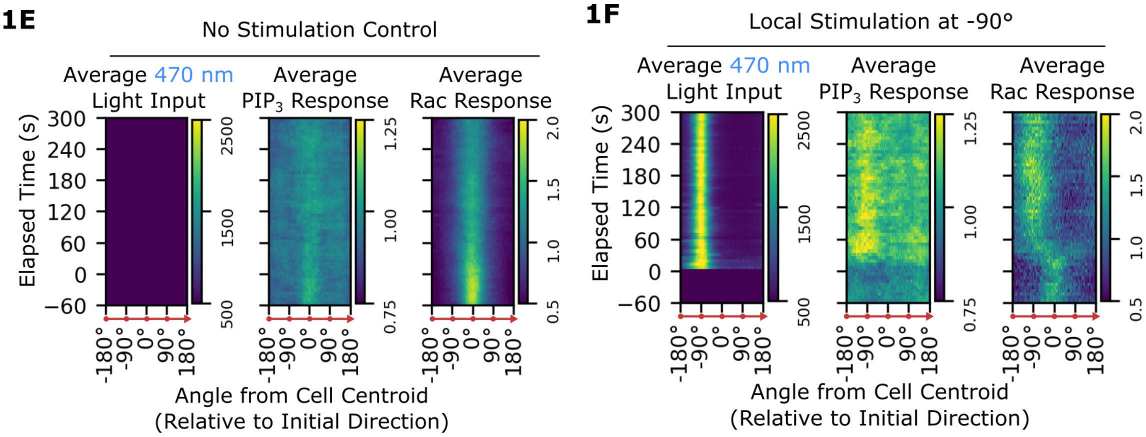

(1E) In the absence of blue-light stimulation, migrating cells maintain their current axis of polarized signaling. These kymographs represent an average of the radial distribution of reflected blue light (Average Light Input, arbitrary units) or normalized fluorescence intensities (Average Normalized PIP_3 Response, Average Normalized Rac Response) over time across all measured no-stimulation control cells (as shown in C, upper-right). Peripheries are aligned so that the initial direction of the cell is 0°.
(1F) In the local stimulation condition, cells reorient both their directionality (as shown in 1D) as well as their biosensor distributions. Like 1E, these kymographs show the averaged radial distribution of measured quantities around the peripheries of migrating cells. There is a local increase in PIP_3 at the -90° location, corresponding to the site of optogenetic stimulation.

4. Experiments in Fig. 2D and G rely on the technical ability to generate PIP3 at two spots on the membrane separately and simultaneously at similar levels. The authors need to show PIP3 biosensor localization in such an experiment to demonstrate technical feasibility,

The raw biosensor data is somewhat noisy in single migrating cells due to the challenges of mapping distributions to the cell frame of reference for our shapeshifting cells. But by averaging over multiple cells, we now show that the average initial local PIP_3 increases do not differ between the winning and losing sides in the 180-degree opposed spot assays (**Current Fig 2B**),

so they likely represent the outcomes of stochastic events rather than biases in our optogenetic experiments.

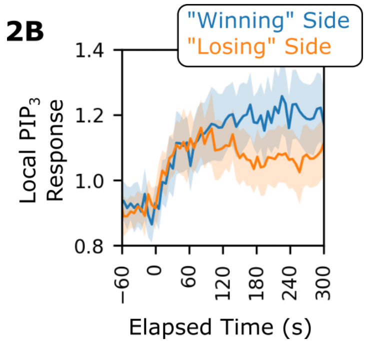

(2B) Local PIP_3 biosensor dynamics are initially similar whether a cell ends up orienting toward a particular stimulation site ("Winning" Side) or away from it ("Losing" Side). Mean local responses are shown with 95% CI shaded.

Minor Concerns:

1. The flow of the figures and the text is occasionally confusing. For example, the text jumps from Fig 2D to 2G before mentioning the figures in between. Additionally, figure 1 is not referenced in the main text.

We have adjusted the flow of the text and figures to be consistent throughout the revised manuscript.

2. There are a few places in the text (like line 64) with extra spaces and/or punctuation. Additionally proofreading would improve the clarity of the manuscript.

We have proofread the current text carefully to improve the clarity of the manuscript.

Reviewer #2: This manuscript characterises spatiotemporal negative feedback mechanisms between PI3K and Rac that control polarisation in DMSO-differentiated HL-60 cells, and presents a new model of this mechanism. Cell biology experiments are presented to support the conclusions. The question being addressed is one that has been discussed, experimented upon and modelled for years by a number of research groups. The new knowledge created might therefore be considered incremental in nature.

We thank the reviewer for their input and hope that we have satisfied their primary concerns in the revision. We have adjusted the introduction and discussion to emphasize that this work connects Meinhardt-style local inhibition to a pilot pseudopod model of cell guidance.

1. A major issue with this paper lies in the fact that Cdc42 is an important regulator of polarisation in mammalian cells, whereas Rac regulates actin polymerization resulting in

formation of a protrusion, often called pseudopod in migrating single cells. In neutrophils, as elsewhere, Cdc42 is crucial for polarisation (e.g. Yang et al 2016, Nat Cell Biology 18, 191; Szczur et al 2009, Blood 114, 4527), although it was not considered here.

Good point. To clarify the role of Cdc42 in cell polarization and guidance, we have repeated our opto-PI3K experiments in Cdc42 knockout HL-60 cells. Though there are some morphological differences from control cells, Cdc42 knockout cells can still polarize, and opto-PI3K can guide the movement of Cdc42 knockout cells (**Fig S10C**). This is likely to be of interest to others in the migration community, so we thank the reviewer for the suggestion.

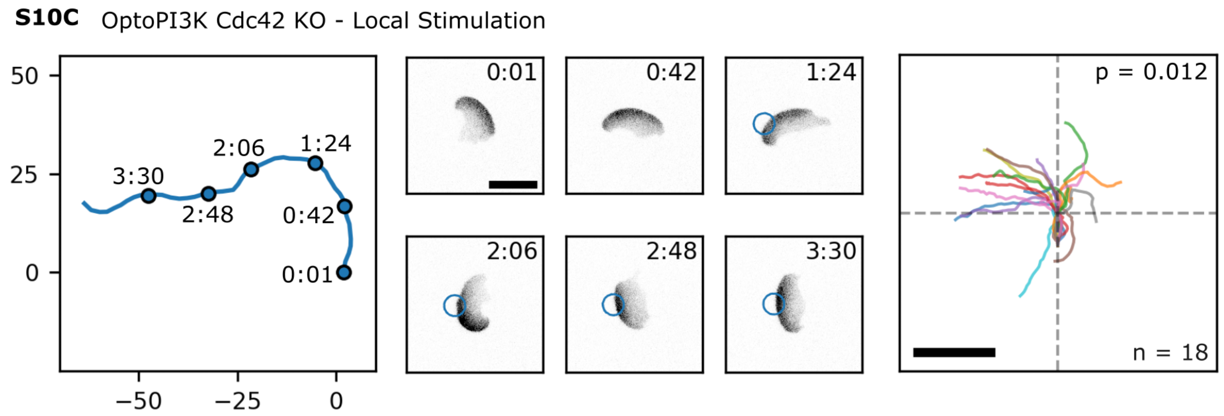

(S10C) In cells that maintain polarization during the course of optogenetic stimulation, local opto-PI3K stimulation retains its ability to steer Cdc42-null HL-60 cells. Cells were stimulated at 1:00 on their left side. The p value refers to the probability that the average final location of cells is unbiased (i.e. is equal to zero). In the presence of a leftward opto-PI3K stimulus, there is a significant bias in cell directionality. Area of optogenetic activation shown with blue outline. Scale bar for microscopy insets (center): 20 µm. Scale bar for migration paths (right): 50 µm.

Unfortunately however the 'biosensor' used in the present manuscript to detect active Rac was Pak-PBD, which is not actually specific for Rac-GTP. Rather it detects both GTP-loaded Rac and Cdc42 and may therefore not differentiate between signals created by these two important regulators. Superior and specific FRET probes have long been described and used (e.g. by Yang et al 2016; Johnson et al 2014 Cell Rep 6, 1153) identifying key roles for both Rac and Cdc42 in single cell chemotaxis. To prove their point, the authors should use such a Rac-specific probe.

While the improved specificity of FRET-based biosensors could be an advantage in many experimental setups, our ability to use these was limited by two factors: 1) the optogenetic system we used is activated by wavelengths of light below 500 nm, so we could not use traditional FRET sensors that operate in that range and 2) Even for the published system from the Collins lab that does operate within an acceptable set of wavelengths, we would then lose the ability to simultaneously monitor PIP_3, limiting our ability to confirm our optogenetic inputs. Thus our choice of the PBD biosensor to read out Rac activation. The reviewer is correct that this probe could be reading out either Rac or Cdc42 activity. To test whether Cdc42 is

significantly contributing to the dynamics of our PBD biosensor, we repeated our optogenetic experiments in Cdc42 knockout cells (**Fig S10**). We find similar adaptive dynamics of the PBD biosensor (**Fig S10A**), confirming this adaptive behavior reflects Rac adaptation and does not depend on Cdc42 binding. Furthermore, our G-LISA experiments confirm that Rac exhibits transient activation downstream of sustained PI3K activation (**Fig S9B**).

S10A

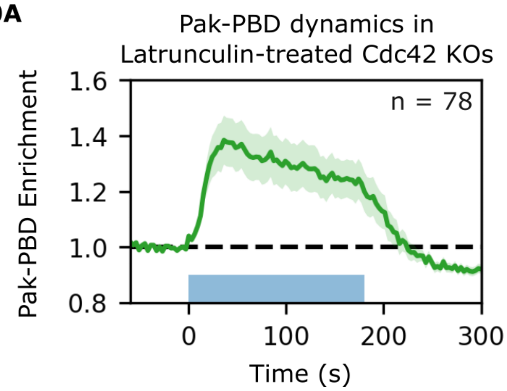

S9B

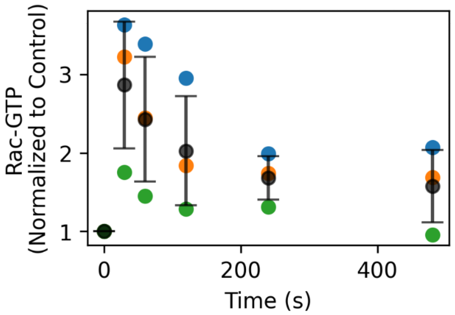

(S10A) Cdc42-null PLB cells exhibit adaptive recruitment of the Rac biosensor PBD following a step input of opto-PI3K, as in wildtype HL-60 cells (compare with Fig 4A, Left). Opto-PI3K-expressing Cdc42-null cells were stimulated with blue light for 180 s, during which time they experienced a decline in the mean Pak-PBD TIRF localization signal. Mean response is shown with 95% CI shaded.

(S9B) GLISA-based measurements of relative Rac-GTP in blue-light-exposed opto-PI3K cells closely match the dynamics observed using the live-cell Rac biosensor, Pak-PBD-mCherry (compare with Fig 4A, Left). Individual replicates are shown in blue, orange, and green. Sample averages ± SD are shown in black.

To demonstrate that PI3K and Rac are the only regulators required for neutrophil polarisation as per the model presented here, their work ought to be complemented with a Cdc42 specific probe; if Cdc42 is indeed dispensable, polarisation should also occur in the absence of Cdc42 activation; this could easily be tested experimentally too.

Good idea. As above, we find that Cdc42 is dispensable for HL-60 polarity, cell guidance by opto-PI3K, and the adaptive dynamics of the PBD biosensor (**Fig S10**).

S10B OptoPI3K Cdc42 KO - No Stimulation

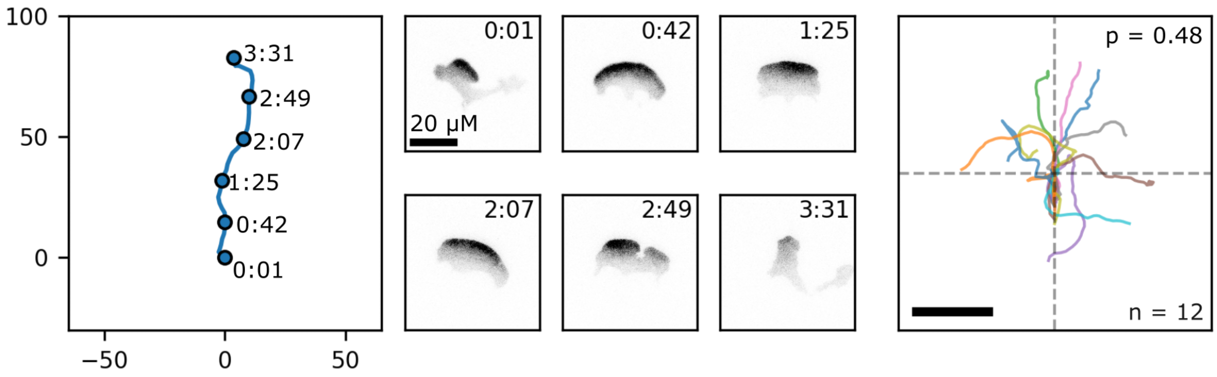

S10C OptoPI3K Cdc42 KO - Local Stimulation

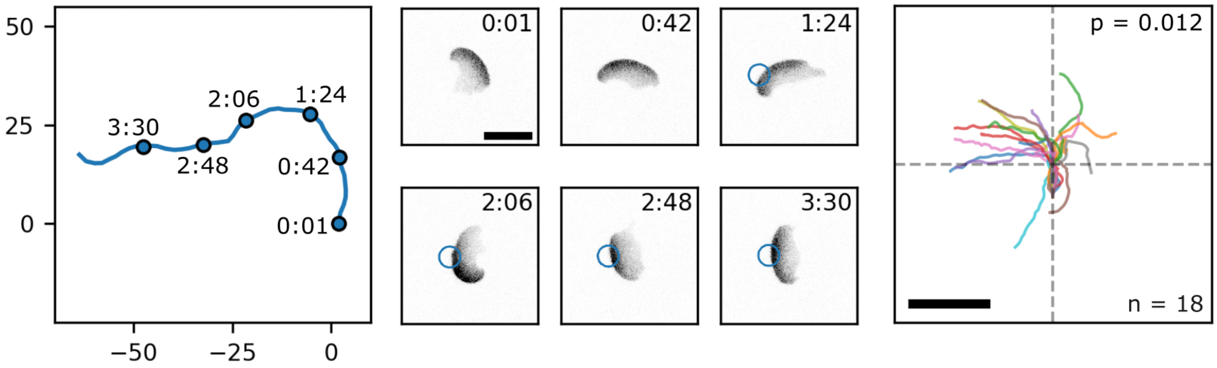

(S10B) Cdc42-null cells maintain their ability to migrate and polarize Pak-PBD. The Cdc42-null cells have broad and relatively unstable fronts that appear to oscillate in width and frequently split. The p value refers to the probability that the average final location of cells is unbiased (i.e. is equal to zero). In the absence of opto-PI3K stimulation, there is no bias. Scale bar for microscopy insets (center): 20 µm. Scale bar for migration paths (right): 50 µm.

(S10C) In cells that maintain polarization during the course of optogenetic stimulation, local opto-PI3K stimulation retains its ability to steer Cdc42-null HL-60 cells. Cells were stimulated at 1:00 on their left side. The p value refers to the probability that the average final location of cells is unbiased (i.e. is equal to zero). In the presence of a leftward opto-PI3K stimulus, there is a significant bias in cell directionality. Area of optogenetic activation shown with blue outline. Scale bar for microscopy insets (center): 20 µm. Scale bar for migration paths (right): 50 µm.

Moreover, since neutrophils (and likely differentiated HL60 cells) express both Rac1 and Rac2 which have overlapping functions in chemotaxis (e.g. Sun et al 2004 Blood 104, 3758), performing actual pull downs with Pak-RBD and probing pull-downs with antibodies specific for Rac1, Rac2 and Cdc42 would be more informative than the G-LISA presented. If G-LISAs are used, a loading control should be shown to ensure that input of the small GTPase in question into all conditions was equal.

We apologize for not being more clear in the methods and have clarified them. The G-LISA assays controlled for cell number so have an equal input concentration for all conditions.

To avoid confusion, the term 'polarisation' should be replaced by alternatives such as pseudopod or protrusion.

We have changed this language where appropriate and have explained our definition of "polarized" in other contexts.

Furthermore, HL-60 cells should not be referred to as 'neutrophils' throughout title, abstract etc. because again this is incorrect and in fact confusing for those who do not know better. Neutrophils are terminally differentiated, primary, short-lived innate immune cells that cannot be cultured nor virally transduced. HL-60 cells are not neutrophils; they would better be described as a model system for amoeboid cell chemotaxis.

We have adjusted our language throughout the manuscript.

Minor points.

1. Much effort appears to have gone into drawing pretty regulatory diagrams (e.g. Fig1) however to a general biologist they are not very clear. The paper would likely reach a wider readership if the network and regulatory diagrams provided were more intuitive to general biologists.

We have adjusted the signaling diagrams (**Current Fig 3A, 3B**) to make them more intuitive to general biologists and removed the smaller network diagrams that other reviewers also found confusing. We hope these new diagrams are more intuitive.

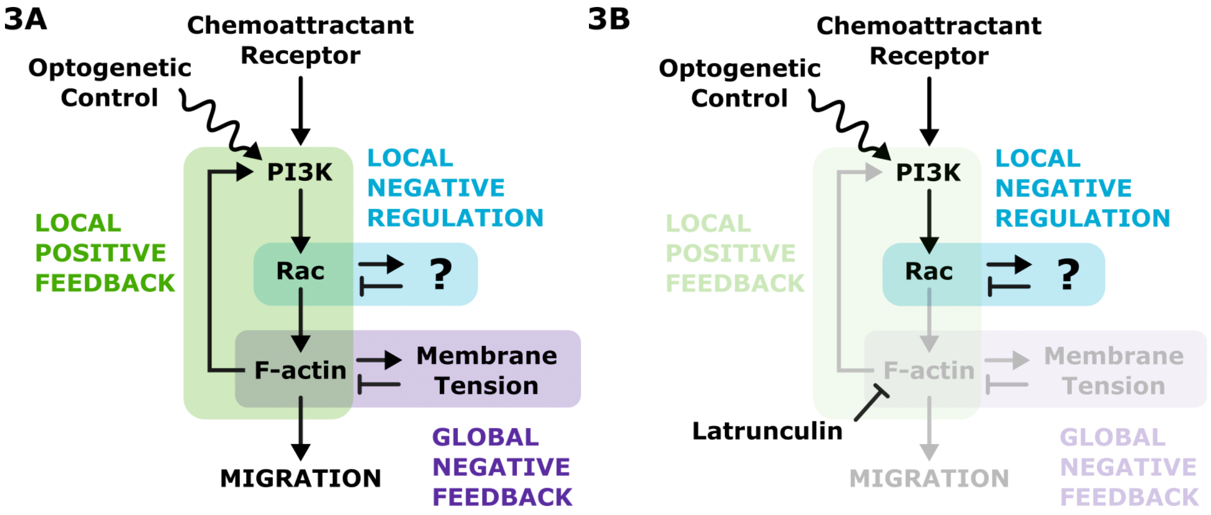

(3A) Cell polarity depends on a combination of positive and negative feedback loops with different spatial ranges of action. Locally-acting positive feedback (green shaded portion, mediated through phospholipids, GTPases, and actin polymerization) locally amplifies the signals that drive cell protrusion. Actin-based protrusions increase the tension in the plasma membrane to globally inhibit secondary sites of protrusion (purple-shaded region). These two

feedback loops (short-range positive, long-range negative) are thought to form the core of a polarity circuit in many cellular contexts but cannot explain the flexibility of cell polarity unless other feedback circuits are also included. An additional locally inhibitory feedback loop has been proposed (blue-shaded region) to avoid the tendency of this core circuit to lock on a given direction of polarity and ignore new stimuli. Direct evidence of this local inhibitor in neutrophil polarity is lacking.
(3B) Because both local positive feedback and global negative feedback are thought to operate through actin-dependent processes, we hypothesized that treating cells with latrunculin B to block actin polymerization would enable us to observe evidence of local inhibition without the confounding effects of the these feedback loops.

2. The methods and figure legends provided are a little scarce. Clearer labelling of the regulatory diagrams in the legends might make them easier to understand. Also information should be provided to indicate on how many separate instances experiments were performed. What was the percentage of cells that moved in these experiments? How often were images acquired during cell migration experiments?

We have expanded on the methods and figure legends to add the suggested details.

3. Line 366-7, cells were isolated by FACS _sorting_

Corrected.

4. Line 490, 2 x 10^5 cells?

Corrected.

5. EHT1864 has been used at 5um in cell based experiments (Shutes et al 2007 JBC 282, 35666), suggesting that 25/50uM doses are not in fact 'mild inhibitions'. An activity assay would identify whether inhibition at the doses used was indeed incomplete. If these doses achieved complete inhibition, an alternative explanation ought to be provided.

The dose of EHT1864 that we used for our experiments is indeed higher than those used in Shutes et al 2007. We have provided a full dose response examining the impact of different doses of EHT1864 on basal Rac activity as indicated by a GLISA assay (**Fig S9C**) and on TIRF-microscopy-based measurements of PAK-PBD dynamics in latrunculin-treated cells (**Fig S9D**). The observed difference between our results and those of the cited work possibly originates in differential permeability or sensitivity in different cell lines - the cited work was tested on 293T cells, while our work was performed in HL-60 cells.

S9C

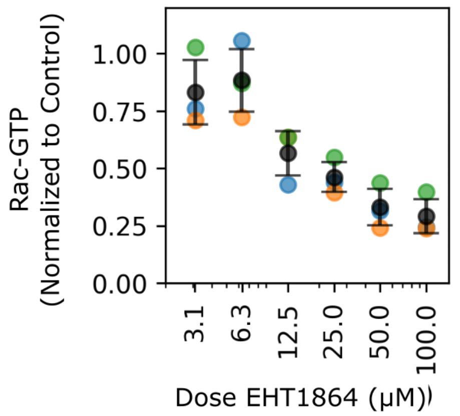

S9D

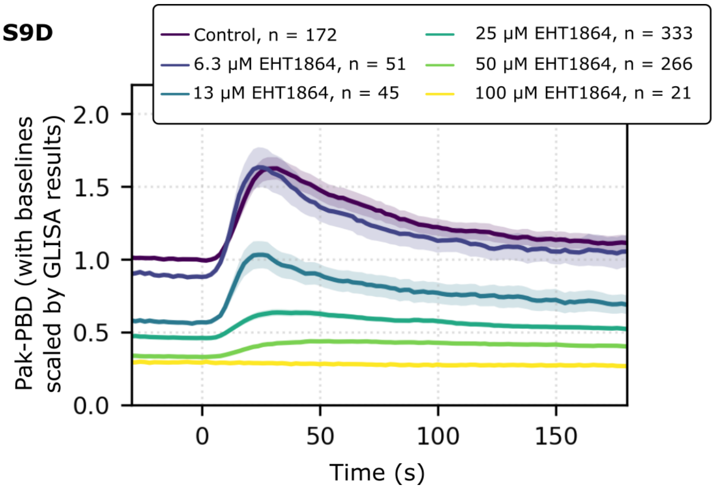

(S9C) Dose-response showing proportion of active Rac in unstimulated opto-PI3K cells (as measured by GLISA) as a function of dose of EHT1864. Individual replicates are shown in blue, orange, and green. Means ± SD are shown in black.

(S9D) Dose response of Pak-PBD dynamics in EHT1864-treated cells. Pak-PBD measurements were made via TIRF microscopy and normalized to baselines that were scaled by GLISA results from S8C. Mean responses are shown with 95 % CI shaded.

Reviewer #3: This excellent study tackles the problem of spatio-temporal signaling in neutrophil chemotaxis. The authors use an optogenetic system to spatio-temporally manipulate PIP3 input signaling, and record PIP3 and Rac signals using biosensors. The big novelty is that they use a computer vision pipeline to do real time image analysis, and program the microscope to provide specific light dependent PIP3 inputs depending on specific signaling states that are a analyzed on the fly. This level of automation allows them to tackle very sophisticated experiments that are very well thought, and that originate on the knowledge that the Weiner lab has accumulated during years of research.

I think that it is fair to say that the technological approach is a tour de force, and that the questions that have been tackled have been extremely well formulated ! This reviewer congratulates the authors for their excellent work.

Now I must admit that I also have some reservations about the results have been spelled out. First, I am not an expert on immune cell chemotaxis, and it was very difficult to navigate the deluge of different chemotaxis models that are the basis for this work that then are crystallized out in the network circuitry in figure 1B ! Given this lack of knowledge, it was difficult for me to completely understand how the findings were able to revise the models. I guess the authors are so familiar with the different models that it is difficult for them to spell out the essence to a lay audience (which I understand is difficult).

We are very pleased that the reviewer is so supportive of our study and realizes all of the foundational work that was necessary to make these experiments possible.

Further, I had the feeling that the figures are very conceptual, and often very succinctly described in the legend. I often had to search the right information about a specific experiment in the text and in the figure legends, which made the manuscript difficult to read. By example the model in figure 1B which depicts a complex feedback circuitry was difficult to understand for me. I missed the AND gate in the text. Maybe the authors could refer to this network in the introduction when they are spelling out the different models, and provide information about each feedback. Also, the network circuitries on the right of figure 2B are not well described in the legends. Another prime example of this is figure 4B. On the network scheme on the left there is a reference to "expected effect of latrunculin". There was no reference to latrunculin in the text. Further, the figure legend relevant to Figure 4B was very conceptual, and did not describe the figure panels ! This applies also to many other figures. I had a bit the feeling that the authors put their conceptual powerpoint slides as figures, but that details about experiments were missing. This made the reading of this manuscript difficult ! I would therefore strongly advise the authors to edit the manuscript to better explain the concepts and the results, so that the paper is smoother to read ! I understand that this will make the paper longer to read.

Great point. We realize that we could have presented the models and circuit diagrams more clearly and have worked to incorporate these suggestions into the revised manuscript. We have adjusted the signaling diagrams (**Current Fig 3A, 3B**) to make them more intuitive to general biologists and removed the smaller network diagrams that the other reviewers also found confusing. We hope these new diagrams are more intuitive.

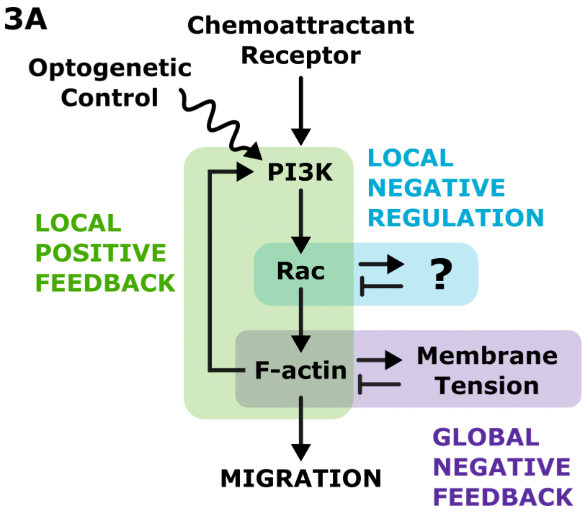

(3A) Cell polarity depends on a combination of positive and negative feedback loops with different spatial ranges of action. Locally-acting positive feedback (green shaded portion, mediated through phospholipids, GTPases, and actin polymerization) locally amplifies the signals that drive cell protrusion. Actin-based protrusions increase the tension in the plasma membrane to globally inhibit secondary sites of protrusion (purple-shaded region). These two

feedback loops (short-range positive, long-range negative) are thought to form the core of a polarity circuit in many cellular contexts but cannot explain the flexibility of cell polarity unless other feedback circuits are also included. An additional locally inhibitory feedback loop has been proposed (blue-shaded region) to avoid the tendency of this core circuit to lock on a given direction of polarity and ignore new stimuli. Direct evidence of this local inhibitor in neutrophil polarity is lacking.
(3B) Because both local positive feedback and global negative feedback are thought to operate through actin-dependent processes, we hypothesized that treating cells with latrunculin B to block actin polymerization would enable us to observe evidence of local inhibition without the confounding effects of the these feedback loops.

Finally, I think that a weakness of the paper is that it lacks a strong modelling component (similar to the work of Iglesias/Devreotes) that can provide better intuition about the results. I think the authors convincingly demonstrate the existence of a negative feedback at the front, but at the same time I feel that the non-linear behavior of such large signaling feedback circuitries can produce signaling behaviors that might escape the intuition of the authors. The modelling part is indeed beyond the scope of this excellent paper. But I would suggest to the authors to mention in the discussion section that this is now the best interpretation of their results, but that mathematical models will allow to refine this. The sophistication and experimental throughput provided by their automated microscopy approach have the power to realize the promise of mathematical modelling.

We completely agree. We have added a remark in the discussion that this would be a valuable future direction. It is possible that some of the emergent features of our cells may already be present in some existing models of cell polarity/movement. Where the models and experiments diverge would help highlight either novel biology or missing features of the models.

I congratulate the authors on their excellent work. I advise them to re-edit the manuscript to make it easier to read, and then I will be ready accept the manuscript !

We thank the reviewer for their enthusiasm and their excellent suggestions for improving the manuscript. We hope they are satisfied with the revision.

Reviewer #4: In response to the cues such as injury or infection, neutrophils polarize their cytoskeletons to migrate directionally. Neutrophils achieve this polarization by amplifying leading edge signals through local positive feedback at the front and global negative feedback. However, these two-component models fail to account for the ability of cells to reorient in response to changing cues. Here, the authors use optogenetics to spatially and temporally control leading edge signaling, a computer-vision-based feedback system, a Rac activity sensor, and modeling to ask how cells polarize and reorient to new cues.

Using an optogenetic PI3K, the authors assess how neutrophils respond to local changes in PI3K activity. They find that cell fronts are more sensitive to than cell backs, and that competing

signals near the fronts of cells can induce direction switching. Using latrunculin A treatment, they demonstrate that Rac is activated locally. But after recovery followed by global activation, Rac activity is lowest where it was previously highest. This leads to the proposal that there is local, temporally delayed, negative feedback.

By inducing fast and slow switches in PI3K activity, they show that Rac responds to the rate of PI3K activation. Further, they show that a prior local stimulation results in a change in direction upon global stimulation. Thus, the data suggest that cells move in the direction of the unstimulated side because this side has a larger temporal increase in stimulus compared to the stimulated side.

The authors thus propose that neutrophils employ local negative feedback onto Rac to prevent "locking" onto a single direction and direct '"peripheral attention" to balance "decisiveness" and "flexibility" during chemotaxis. The study design is creative and the experimental approaches are sophisticated. The results presented are mostly clear, informative, and relevant for readers of PLOS Biology. However, the presentation could be clearer and some attempt to uncover a mechanism driving this local negative feedback would be welcome, as described below.

We thank the reviewer for their support of our findings and approach. We have incorporated their suggestions for improving the manuscript in the revision.

Major:

What kinds of mechanisms do the authors think could be operating? Could it simply be that Rac turns itself off over time by hydrolyzing GTP to GDP? Or does the negative feedback require that PI3K, PIP3, RacGTP, or something else recruit a GAP to accelerate Rac inactivation (as implicated in the text? "Together these data suggest that local PIP3 generation leads to recruitment of a reversible, locally acting, moderately persistent Rac inhibitor." Could the effect be due to local depletion of an activator or a refractory period for an activator, internalization of Rac itself, or multiple mechanisms? Mutations that affect the Rac GTPase activity or its interaction with GAPs could address some of these possibilities. Even negative results might be interesting if they rule particular possibilities out. If this point is beyond the scope of this study, the authors should not refer to a discrete inhibitor because the nature of the inhibition is completely unknown. Some discussion of possible mechanisms would be helpful as well.

Good point. We are much more explicit in the revised manuscript with regards to possible mechanisms of local inhibition and are more clear that our findings are consistent with local inhibitor(s). We performed a focused candidate knockout screen for some of the obvious molecular candidates for local inhibition (ARHGAP9, ARHGAP12, ARHGAP15, ARHGAP24, ARHGAP35, ARHGAP41, MYO9B, CHN2, and CYRI-B), but none of these yielded our expected phenotype (sustained, rather than transient Rac activation in response to optogenetic stimulation), either because these are the wrong candidates or possibly due to redundancy between multiple local inhibitors. Because we did not confirm genetic ablation for each candidate in a clonal fashion, we did not include these negative results in the manuscript.

The main experiment showing a reduction in Rac activity at the initial site of activation upon global stimulation is with Latrunculin A treatment. They state that global tension mediated negative feedback might be difficult to disentangle from a local negative inhibitor. However, it also seems important to assess the contribution of the local inhibition in the context of the more dominant global negative feedback in a normally migrating cell. Can the authors look at the Rac activity reporter as they do with the PIP3 sensor in the assay used in Figure 5? What happens to Rac activity in this context?

This is a really nice thought. We might have expected the Rac responses on each side to look very similar to the derivative of the PIP_3 profiles shown in Fig 5E. We did try this and the result is not very satisfying (not included in the manuscript, but see below). The reason for this is that the Rac biosensor polarity is nearly instantaneously correlated with cell directionality in migrating cells. So in the end, we used directionality as our readout rather than the Rac distribution in this context.

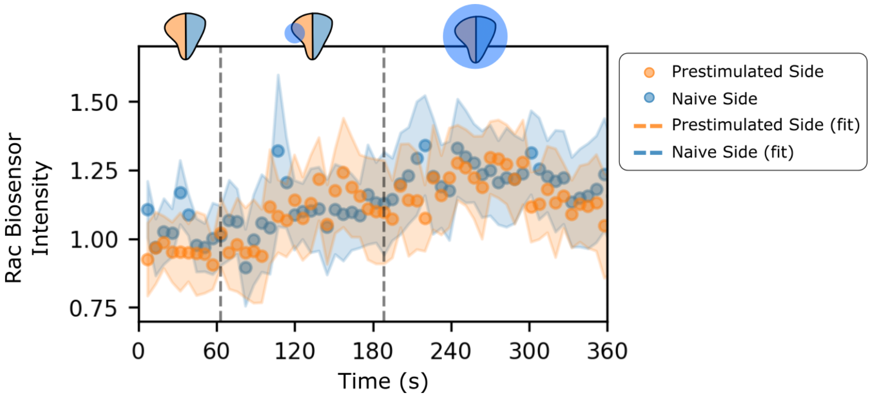

The biosensor spatiotemporal dynamics appear to be different depending on the whether the PI3K stimulus was provided at the front, side or back. Have the authors quantified this distribution and whether this affects cell polarization? For example: When the PI3K stimulus was provided at the back of the cell (Figure 2C), the biosensor polarization at the front was not disrupted over several minutes whereas when the stimulus was provided on the side, the turning appeared rapid.]

This is true and is consistent with what other researchers in the field have seen. Positive feedback tends to keep the fronts stable, so small reorientations are generally favored over complete reversals. It is likely an effect of decreased competence for both PIP_3 and Rac signaling at the backs of cells. Similar results were recently reported by the Theriot lab (PMID: 34785640).
We have now provided a comparison of turning rates for the different local stimuli (**Fig S2D**). One interesting observation from this quantification is that back-stimulated cells don't appear to accelerate their alignment when they are half-way through their turns to resemble the alignment kinetics of side-stimulated cells. This is likely due to the rate-dependence of the response. The PIP_3 levels have already started to increase when these cells are half-way through their 180° reversal.

S2D

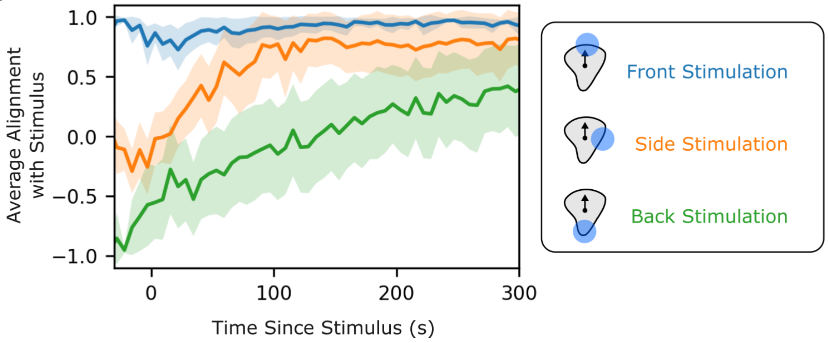

(S2D) The angles of displacement for each cell were calculated and cosine of the difference between these angles and the angle of stimulus were then calculated for each assay type as an indicator of stimulus alignment dynamics. Interestingly, back-stimulated cell alignment dynamics do not appear to match side-stimulated dynamics, even when they are spatially identical (i.e. when the green curve passes 0 on the y axis). This is likely due to the spatial insensitivity at the cell back and the resulting slow local accumulation of PIP_3 in this assay. Mean responses are shown with 95 % CI shaded.

It would be helpful to show the actin dynamics in LatA treatment and the shadow experiments.

Based on the amount of latrunculin used and the lack of protrusions in microscopy data from Figure 3 (**Previous Fig 3C, Current Fig 3D**), we don't expect any actin dynamics in this experiment. Unfortunately, we did not have a live-cell actin reporter present in these cells, but these latrunculin conditions are sufficient to block the global inhibitor (**Fig. 3C, S7**), which is the primary purpose of this perturbation. This dose of latrunculin has also been used in previous studies from our lab, where it was shown to be sufficient to block actin assembly (PMID: 17696648, PMID: 37311454).

3C

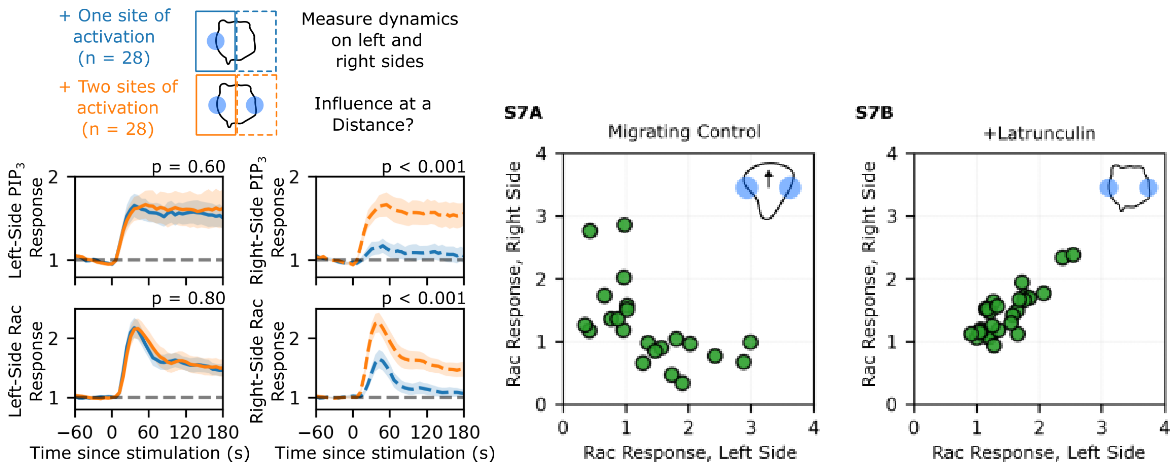

(3C) To demonstrate the absence of global inhibition in latrunculin-treated cells, we exposed them to either one or two spots of opto-PI3K activation and compared the resulting dynamics on

both sides. In the absence of a global inhibitor, the responses for a single opto-PI3K stimulus should be the same whether or not there is a secondary site of opto-PI3K activation (in contrast to competition between these sites in the presence of the global inhibitor, as in Fig 2A-C). When the right sides of cells are compared in each type of stimulation, the presence or absence of the local right stimulus makes a large difference in the observed signaling dynamics. However, the dynamics on the left sides of cells do not differ based on the presence or absence of a distant site of stimulation. Thus, there is no inhibitory action-at-distance, which would be expected from a global inhibitor. The p values above each graph indicate the probability of observing an absolute difference greater than or equal to that observed by a permutation test. Mean local responses are shown with 95% CI shaded.

(S7) By comparing the Rac biosensor dynamics on the left and right sides of cells, we tested for the winner-take-all behavior expected in the presence of a global inhibitor. Each point is the relative Rac enrichment (compared to baseline) at the end of a 5-minute two-spot opto-PI3K activation assay. **(A)** In control cells stimulated with opto-PI3K at both their left and right edges (relative to their initial directionality), one site becomes Rac-high and one becomes Rac-low, leading to anticorrelated Rac distributions between the two sides. **(B)** In latrunculin-treated cells stimulated on both their right and left edges with opto-PI3K, this winner-take-all system is not functional, and the two sides now show a positive correlation of Rac activity levels.

In Figure 3C, what would the Rac biosensor look like with a two-spot assay stimulation at the lateral edges after recovery? Can the authors use the model to predict the outcome in such an experimental scenario and then test the prediction?

We would expect this to be very similar to what we observe with the local-to-global shadow experiments **(Fig 3D)**. The primary difference would be the total area of the cell surface activated.

3D

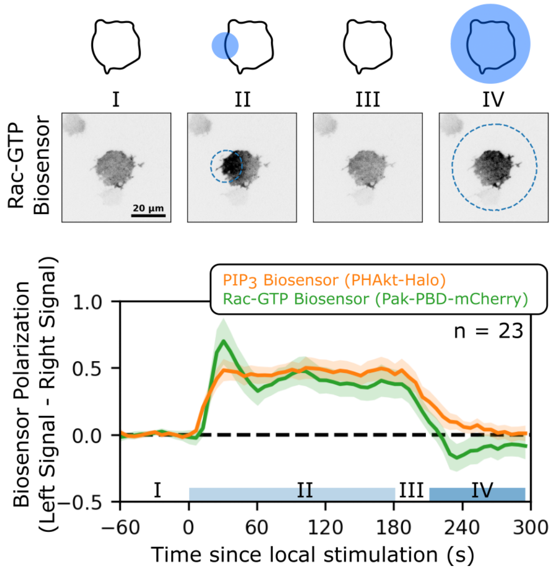

(3D) To demonstrate the presence of local inhibition, we first exposed single cells to a local opto-PI3K stimulus on their left sides, then allowed cells to recover briefly, and then exposed them to a global opto-PI3K stimulus. We reasoned that if the decline in signaling on the left side were due to a local inhibitor, the impact of that inhibition should be observable via a weaker response on the prestimulated side. We observed this evidence of local inhibition at the level of Rac activation (green – Rac is biased towards the right side in phase IV) but not at the level of the opto-PI3K input (orange – no bias of PIP_3 to the right side in phase IV). Mean local responses are shown with 95% CI shaded.

Have the authors noticed differences in protrusion lifetimes in the shadow experiments in Figure 3C and 5B?

In our conceptual model, the role of local inhibition is to enable spatial fine-tuning of the position of the protrusion using local temporal information (**Fig 6, right**) rather than to set the protrusion lifetime, as expected from a classical pilot pseudopod model (**Fig 6, center**). In Figure 3C (**Previous Fig 3C, Current Fig 3D**), cells were treated with 10 μM latrunculin, so they are not capable of generating protrusions and lack global inhibition. Thus, we don't expect the timescales of the two conditions to be directly comparable.

Minor:

Figure 1 is not cited in the text and is purely conceptual. While helpful, could it be condensed and perhaps merged with a figure that contains data?

Excellent point. As suggested, we have moved the conceptual overview into the supplement, and we agree that this is a much better way to start the manuscript.

Line 103, pg4: The authors go back and forth between referring to Fig2G and 2D before E and F. The figures should be arranged and discussed in order.

In the revised manuscript, all figures are now ordered by first appearance in the text.

Figure 3A needs to be labeled- the reader cannot interpret this with figure or figure legend

We realize that we could have presented the models and circuit diagrams more clearly and have worked to incorporate these suggestions into the revised manuscript. We have adjusted the signaling diagrams (**Current Fig 3A, 3B**) to make them more intuitive to general biologists and have removed the smaller network diagrams that the other reviewers also found confusing. We hope these new diagrams are more intuitive.

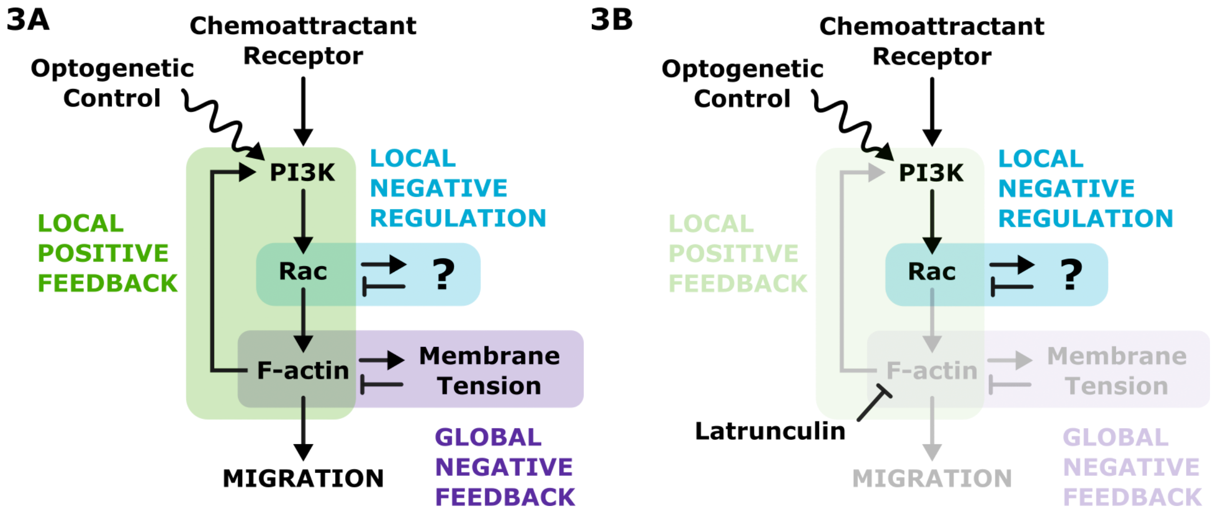

(3A) Cell polarity depends on a combination of positive and negative feedback loops with different spatial ranges of action. Locally-acting positive feedback (green shaded portion, mediated through phospholipids, GTPases, and actin polymerization) locally amplifies the signals that drive cell protrusion. Actin-based protrusions increase the tension in the plasma membrane to globally inhibit secondary sites of protrusion (purple-shaded region). These two feedback loops (short-range positive, long-range negative) are thought to form the core of a polarity circuit in many cellular contexts but cannot explain the flexibility of cell polarity unless other feedback circuits are also included. An additional locally inhibitory feedback loop has been proposed (blue-shaded region) to avoid the tendency of this core circuit to lock on a given direction of polarity and ignore new stimuli. Direct evidence of this local inhibitor in neutrophil polarity is lacking.
(3B) Because both local positive feedback and global negative feedback are thought to operate through actin-dependent processes, we hypothesized that treating cells with latrunculin B to block actin polymerization would enable us to observe evidence of local inhibition without the confounding effects of the these feedback loops.

Panels 3D and 3E are not cited in the text.

We have made sure that we cite every subpanel in order in the text in the revised manuscript.

Figure 5E - were these measurements performed on multiple experimental replicates? This should be shown to show the variability between experiments.

We have now included a 95% confidence interval for the data (**Fig 5E**).

5E

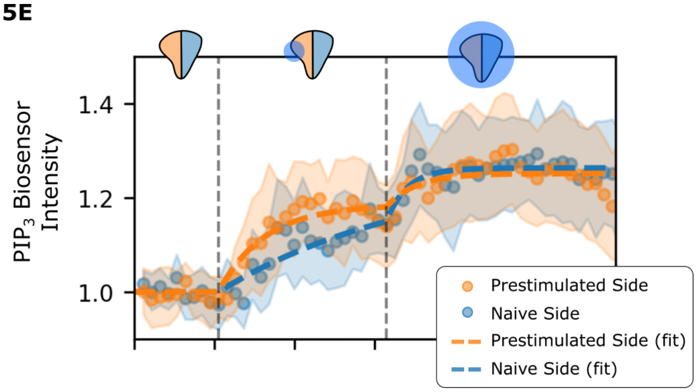

(5E) The PIP_3 signals on the left and right sides of migrating cells were calculated and then fit with a time-delayed saturable function. Sample means are shown as dots, and the 95 % confidence interval is shown as the shaded area. The fit functions are shown as dashed lines. As expected, both sides approach the same steady state at the end of phase III, but their histories differ. The changes in cell directionality correlate with the local derivative of input signals rather than the absolute levels of the input signal, consistent with a pilot pseudopod model of gradient interpretation.

Statistical testing and P values should to be added to some figures (specifically noticed Figures 2D, and S5)

We have added more statistical tests throughout the manuscript where appropriate.

Is there a way to test if the inhibition at one site occurs during reorientation/direction switching of neutrophils without optogenetics?

We unfortunately don't have a way to deliver inputs in our lab with sufficient spatial precision for two spot assays without optogenetic inputs.

Can the authors think of a way to shorten the title to make it clearer? For example "Rac negative feedback" is not very clear. Negative feedback from what to what?

We have revised the title to: Local negative feedback underlies a pilot pseudopod-like program for amoeboid guidance
