# Supplementary material for: Local negative feedback of Rac activity at the leading edge underlies a pilot pseudopod-like program for amoeboid cell guidance
Source: PLoS Biol. 2023 Sep 25;21(9):e3002307. doi: 10.1371/journal.pbio.3002307 (PMC10553818; doi:10.1371/journal.pbio.3002307)
Supplement: Attachment — Submitted filename: point.by.point.jason.v3.docx [file pbio.3002307.s021.docx]

**Point-by-point response to Editors and Reviewers**

(A) We would like to suggest the following modification to the title:
“"Local negative feedback of Rac1 activity at the leading edge underlies a pseudopod-like program for amoeboid cell guidance”

Great suggestion—we have modified it slightly to indicate Rac instead of Rac1 (likely both Rac1 and Rac2 are involved, and our PBD probe does not distinguish) and to include a pilot pseudopod-like program. Our revised title is:

"Local negative feedback of Rac activity at the leading edge underlies a pilot pseudopod-like program for amoeboid cell guidance"

(B) Thank you very much for already providing the underlying numerical data for the figures presented in the manuscript. However, we note that Fig S9A appears to be mislabeled in the data file, as this refers to a cartoon diagram in the manuscript?

Good catch. We have corrected that in the updated data file spreadsheet.

(C) Per journal policy, as the code that you have generated is important to support the conclusions of your manuscript, we require that you make it available without restrictions upon publication. Please ensure that the code is sufficiently well documented and reusable, and that your Data Statement in the Editorial Manager submission system accurately describes where your code can be found. If depositing code on Github, we ask that you please attach this to Zenodo to ensure long term maintenance and that the deposition is assigned a DOI.

We have uploaded as requested to Zenodo and have included the DOIs in the paper.

- The data is located at: [https://doi.org/10.5281/zenodo.8208724](https://urldefense.com/v3/__https:/doi.org/10.5281/zenodo.8208724__;!!LQC6Cpwp!qNA2tBBfsdDWKbWl-DOo_q4r299FktTZXqd6E0dPBzci7XBhX_wkegx2iMWXiW3Q0nVkaUFl7APIIwI7oiHy3vninQ$)
- The analysis notebooks are at: [https://zenodo.org/record/8217762](https://urldefense.com/v3/__https:/zenodo.org/record/8217762__;!!LQC6Cpwp!qNA2tBBfsdDWKbWl-DOo_q4r299FktTZXqd6E0dPBzci7XBhX_wkegx2iMWXiW3Q0nVkaUFl7APIIwI7oiEgY40KDg$)
- And the microscopy automation code is: [https://zenodo.org/record/8217768](https://urldefense.com/v3/__https:/zenodo.org/record/8217768__;!!LQC6Cpwp!qNA2tBBfsdDWKbWl-DOo_q4r299FktTZXqd6E0dPBzci7XBhX_wkegx2iMWXiW3Q0nVkaUFl7APIIwI7oiGOXZywnw$)

(D) Please also ensure that each of the relevant figure legends in your manuscript include information on *WHERE THE UNDERLYING DATA CAN BE FOUND*, and ensure your supplemental data file/s has a legend.

We have added a short phrase to the end of each figure legend (except Fig 6 which is purely conceptual) indicating that the numerical data is available in S1 Data.

(E) Please ensure that your Data Statement in the submission system accurately describes where your data can be found and is in final format, as it will be published as written there. As noted before, this includes the deposition for the code.

We have included the location of the data, analysis notebooks, and microscopy automation code in Zenodo in the Data Statement.

(F) Please ensure that the funders and grant numbers match between the Financial Disclosure field and the Funding Information tab in your submission form. Note that the funders must be provided in the same order in both places as well.

The funder numbers and order are now consistent between the Financial Disclosure field and the Funding Information tab.

(G) Please upload your main article file as a .doc, .docx or .rtf file.

We have uploaded our main article as a .docx file as well as a pdf file in the PLoS Biology template.

(H) Your Response to Reviewers should be uploaded as a single, individual file. Please remove your Response to Reviewers from your Cover Letter."

We have removed the Response to Editors/Reviewers from the Cover Letter and have now included it as its own file.
